# Infectious Complications of CD19-targeted Chimeric Antigen Receptor T-cell Therapy: a Multicenter Cohort Study

**DOI:** 10.1093/ofid/ofag423

**Published:** 2026-07-03

**Authors:** Jonathan Huggins, Julia A Messina, Jennifer Saullo, Jiayi Xie, Amy Zhang, Ishan Paranjpe, Rong Lu, Megan Walsh, Michael Mohnasky, Joseph Stromberg, Felicia Cao, Natalie Grover, Manish Saha, Alicia Darwin, Nancy Torres, Patrick Tam, Daniel Schrum, Erin Eberwein, Erin Kennedy, Christopher R Kelsey, Taewoong Choi, Matthew McKinney, Yubin Kang, Melody Smith, Tessa M Andermann

**Affiliations:** Division of Infectious Diseases, Department of Medicine, Duke University Medical Center, Durham, North Carolina, USA; Division of Infectious Diseases, Department of Medicine, Duke University Medical Center, Durham, North Carolina, USA; Division of Infectious Diseases, Department of Medicine, Duke University Medical Center, Durham, North Carolina, USA; Division of Blood & Marrow Transplantation and Cellular Therapy, Department of Medicine, Stanford University, Stanford, California, USA; Department of Medicine, Quantitative Sciences Unit, Stanford University, Stanford, California, USA; Division of Blood & Marrow Transplantation and Cellular Therapy, Department of Medicine, Stanford University, Stanford, California, USA; Department of Medicine, Quantitative Sciences Unit, Stanford University, Stanford, California, USA; Department of Medicine, University of North Carolina at Chapel Hill, Chapel Hill, North Carolina, USA; Department of Medicine, University of North Carolina at Chapel Hill, Chapel Hill, North Carolina, USA; Department of Medicine, University of North Carolina at Chapel Hill, Chapel Hill, North Carolina, USA; Department of Medicine, University of North Carolina at Chapel Hill, Chapel Hill, North Carolina, USA; Department of Medicine, University of North Carolina at Chapel Hill, Chapel Hill, North Carolina, USA; Department of Medicine, University of North Carolina at Chapel Hill, Chapel Hill, North Carolina, USA; Department of Medicine, Stanford University Hospital, Stanford, California, USA; Department of Medicine, Stanford University Hospital, Stanford, California, USA; Division of Infectious Diseases, Department of Medicine, Duke University Medical Center, Durham, North Carolina, USA; Division of Hematologic Malignancies and Cellular Therapy, Duke University Medical Center, Durham, North Carolina, USA; Division of Hematologic Malignancies and Cellular Therapy, Duke University Medical Center, Durham, North Carolina, USA; Division of Hematologic Malignancies and Cellular Therapy, Duke University Medical Center, Durham, North Carolina, USA; Department of Radiation Oncology, Duke University Medical Center, Durham, North Carolina, USA; Division of Hematologic Malignancies and Cellular Therapy, Duke University Medical Center, Durham, North Carolina, USA; Division of Hematologic Malignancies and Cellular Therapy, Duke University Medical Center, Durham, North Carolina, USA; Division of Hematologic Malignancies and Cellular Therapy, Duke University Medical Center, Durham, North Carolina, USA; Division of Blood & Marrow Transplantation and Cellular Therapy, Department of Medicine, Stanford University, Stanford, California, USA; Division of Infectious Diseases, Department of Medicine, University of North Carolina at Chapel Hill, Chapel Hill, North Carolina, USA

**Keywords:** chimeric antigen Receptor T-cell, lymphoma, immunocompromised host, CD19, bacterial, fungal, viral

## Abstract

**Background:**

Our understanding of the epidemiology of and risk factors for postchimeric antigen receptor T-cell therapy (CART) infections is largely based on data from single-center studies with small sample sizes.

**Methods:**

This is a multicenter, cohort study of adult patients treated with CD19 CART between 1 January 2018 and 31 August 2021. The epidemiology of infectious complications occurring within the first year after CART is described. A Fine-Gray subdistribution hazard model was built to identify risk factors for infection. Logistic regression was used to explore risk factors for early (≤90 days post-CART) versus late (>90 days post-CART) and bacterial versus viral infection.

**Results:**

One hundred and thirty-one infections occurred in 97 of 311 patients (31.2%) within 1 year of CART. By infection type, the cumulative incidence of infection was 19.0% (viral), 20.9% (bacterial), and 2.3% (fungal). The majority of infections were mild or moderate in severity (86%). *Clostridiodes difficile* and bloodstream infections due to Gram-negative bacilli were common bacterial infections. Respiratory viral infections were the most common viral complication and fungal infections were rare. No independent predictors of infection were identified.

**Conclusions:**

Infectious complications occur in close to a third of patients post-CART but are generally mild in severity. Though we were not able to identify independent predictors of post-CART infection, a description of their clinical characteristics and epidemiology will help to optimize management of these common complications.

## Teaser

Post-CD19 chimeric antigen receptor T-cell therapy infections were found to be common, but most were mild or moderate in severity. *Clostridioides difficile*, Gram-negative bacilli bloodstream, and respiratory viral infections were common. Fungal infections are rare but may have severe outcomes.

CD19-directed chimeric antigen receptor T-cell therapy (CART) has fundamentally altered the treatment of refractory B-cell malignancies. Despite their high efficacy, these therapies are accompanied by “on-target, off-tumor” effects that, in addition to antecedent lymphodepleting (LD) chemotherapeutic regimens, contribute to the already considerable net state of immunosuppression in this patient population. As a result, infection is a common and sometimes life-threatening complication of CART [1–5].

Multiple reports have described infectious complications following CART but have important limitations [6–19]. Most are single-center retrospective studies and their results may not be generalizable to a broader population since center-to-center variation in antimicrobial prophylaxis practices and geographic differences in epidemiology may affect infection outcomes. Further, study-to-study variation in the definition of infection complicates the interpretation of results. Because few prior studies have followed patients for a year post-CART, late infections are less well-described [6, 8, 12, 13, 16–18].

This study endeavors to address the limitations of the existing literature through its multicenter design, larger sample size, and comprehensive definitions of infection. We included all adult patients who underwent CD19-directed CART at Stanford University (SU), Duke University (DU), or the University of North Carolina—Chapel Hill (UNC) between 1 January 2018 and 31 August 2021. Patients were followed up to 1 year following CART to determine rates of and risk factors for bacterial, fungal, viral, and parasitic infections.

## METHODS

### Design

This is a retrospective, multicenter cohort study including patients who received CD19-directed CART at SU, DU, or UNC between 1 January 2018 and 31 August 2021. Infectious outcomes were evaluated between the time of CAR T-cell infusion and death, loss to follow-up, receipt of additional chemotherapy, or 1 year post-CART. The study was conducted in accordance with the Declaration of Helsinki and independently approved by the DU, SU, and UNC Institutional Review Boards.

### Population

The study included all adult patients (≥18 years of age) who received CD19-directed CART for the management of a B-cell malignancy at one of the three participating institutions during the study timeframe. In patients who received a second CAR T-cell infusion, only the first event was evaluated. Patients were excluded if they were <18 years of age or received CART at an outside institution.

### Institutional Management Protocols

Supplementary Table 1 displays the prophylaxis protocols at each study site. Antibacterial, antiviral, and *Pneumocystis jirovecii* prophylaxis were applied at all three institutions. Whereas DU and UNC provided fluconazole prophylaxis, SU did not. At SU and UNC, routine immunoglobulin G (IgG) level monitoring was recommended. UNC recommended administration of intravenous immunoglobulin (IVIg) for IgG values <400 mg/dL in patients with serious or recurrent infections. IgG monitoring and IVIg administration at DU were at the providers’ discretion. Growth colony-stimulating factor (GCSF) was administered daily to all patients at SU (except those receiving tisagenlecleucel) starting on day +1 after CAR T-cell infusion until the absolute neutrophil count (ANC) was >1000 cells/µL. At UNC and DU, GCSF was administered at the providers’ discretion.

### Objectives

Our primary objective was to describe the 1-year incidence of infection among recipients of CD19-directed CART both overall and by pathogen type (viral, bacterial, fungal, or parasitic). We also describe infection incidence by previously identified risk periods (0–28 days post-CART, 29–90 days post-CART, and 90–365 days post-CART) [2, 3, 5].

Secondary objectives included describing the microbiology of post-CART infections, comparing the demographic and clinical characteristics of patients by infection status, describing clinical outcomes by infection status, and identifying risk factors for infection within the first year after CART. As exploratory aims, we evaluated risk factors for infection by pathogen type and for early (≤90 days post-CART) and late (>90 days post-CART) infection.

### Data Collection and Definitions

Demographic and clinical variables were manually abstracted from the electronic health record by investigators at each institution. Definitions of bacterial and viral infection are detailed in the Supplementary Appendix. With the exception of herpes zoster and herpes simplex infection, which could be diagnosed based on characteristic skin lesions, we required microbiological confirmation of infection events. Clinically relevant cytomegalovirus (CMV) infection was defined as CMV DNA detection in the serum that resulted in initiation of antiviral therapy, or that occurred in the context of a CMV syndrome or CMV disease as defined in the Supplementary Appendix. Fungal infections were included according to proven or probable criteria established by the European Organization for Research and Treatment of Cancer and the Mycoses Study Group [20]. Infection severity was defined according to the Blood and Marrow Transplant Clinical Trials Network (BMT-CTN) grading system [21]. Grading of cytokine release syndrome (CRS) and immune effector cell-associated neurotoxicity syndrome (ICANS) was in accordance with the American Society for Transplantation and Cellular Therapy (ASTCT) consensus criteria [22]. The CAR HEMATOTOX Score was calculated as described by Rejeski et al [23]. Neutropenia was defined as ANC of <0.5 × 10^9^ cells/L. Lymphopenia was defined as an absolute lymphocyte count of <0.2 × 10^9^ cells/L.

Medication administration records were used to measure duration of antimicrobial exposure. If administration data were not available, prescription beginning and end dates were used. Perfect compliance was assumed. All antimicrobials administered between the date of CART infusion and loss to follow-up, death, initiation of chemotherapy for relapsed disease, or 1-year post-CART were included. Immunosuppressive exposure was collected in an analogous fashion. All immunosuppressive agents excluding chemotherapeutics for relapsed disease were considered, including those used to treat CRS and ICANS.

### Statistical Analysis

Baseline characteristics and clinical outcomes were compared by infection status using χ^2^ and Wilcoxon rank-sum tests for categorical and continuous data, respectively. To evaluate risk factors for post-CART infection, a time-to-event analysis using a Fine-Gray subdistribution hazard function was performed with death as a competing risk and censoring at loss to follow-up and receipt of additional chemotherapy. A marginal screening threshold of *P* ≤ .05 in the univariate analysis was used for entry into an age- and disease-adjusted multivariate model. Antimicrobial and immunosuppressive exposure were handled as time varying covariates. Logistic regression was used to assess for independent risk factors for early infection (≤90 days post-CART), late infection (>90 days post-CART), bacterial infection, and viral infection. A marginal screening threshold of *P* ≤ .05 in the univariate analysis was used for entry into an age- and disease-adjusted multivariate model. If both the CAR HEMATOTOX score and its components met the univariate logistic regression screening threshold, CAR HEMATOTOX was excluded from the multivariate analysis to avoid issues arising from collinearity. The total duration of antimicrobial or immunosuppressive exposure was considered only up to the date of the infectious outcome of interest. While the resulting variation in exposure period among patients with or without incident infection during follow-up introduces a risk of ascertainment bias, we considered the limitation acceptable given the exploratory nature of the analysis and the fact that any bias would likely be toward the null hypothesis. All analyses were performed using Stata 18.0 or SAS, version 9.4.

## RESULTS

### Patient Demographics and Treatment Characteristics

Between 1 January 2018 and 31 August 2021, 311 patients underwent CART at SU (n = 195), DU (n = 66), or UNC (n = 50). The majority of patients (62.7%) were male and White (66.9%) with underlying diffuse large B-cell lymphoma (DLBCL) (77.5%). Ninety-six patients (30.9%) had previously undergone hematopoietic cell transplantation (HCT) (Table 1). A majority of patients (71.4%) received Axicabtagene ciloleucel (Table 1). Most patients (83.9%) experienced CRS. A majority of cases (97.0%) were Grade 1 or 2 and 72.8% of patients were treated with immunomodulatory therapies. ICANS was observed in 45.3% of patients and 41.1% of these cases were ≥ Grade 3. A majority of patients with ICANS received treatment (93.6%) (Table 2). Over a third (39.9%) of patients had relapse of their underlying malignancy within a year of CART, and an additional 12.9% never responded to CART. One-year all-cause mortality was 25.7% (Table 1).

**Table 1.** Demographic, Disease and Treatment Characteristics of Patients Receiving CD19-directed Chimeric Antigen Receptor T-cell Therapy at Stanford University, Duke University, or University of North Carolina—Chapel Hill

Factor	Level	Value
*N*		311
Study site	Stanford	195 (62.7%)
	UNC	50 (16.1%)
	Duke	66 (21.2%)
Age, median (IQR)		63 (52, 70)
Sex	Female	116 (37.3%)
Race	African descent	23 (7.4%)
	Caucasian	208 (66.9%)
	Asian descent	32 (10.3%)
	Pacific Islander	3 (1.0%)
	Unknown	9 (2.9%)
	Other	37 (11.6%)
Ethnicity	Hispanic	38 (12.2%)
	Non-Hispanic	266 (85.5%)
	Unknown	7 (2.3%)
CCI, median (IQR)		4.0 (3.0, 5.0)
KPS	50	2 (0.7%)
	60	10 (3.4%)
	70	61 (20.6%)
	80	99 (33.4%)
	90	102 (34.5%)
	100	18 (6.1%)
	Unknown	19 (6.1%)
ECOG performance status	0	70 (27.5%)
	1	151 (59.2%)
	2	29 (11.4%)
	3	1 (0.4%)
	Unknown	60 (19.3%)
Underlying malignancy	DLBCL	241 (77.5%)
	Non-DLBCL NHL	52 (16.7%)
	ALL	17 (5.5%)
	Other	1 (0.3%)
Disease status pre-CART	CR	4 (1.3%)
	PR	37 (11.9%)
	VGPR	3 (1.0%)
	PD	214 (68.8%)
	SD	51 (16.4%)
	Other	2 (0.6%)
Number of prior lines of chemotherapy, median (IQR)		3.0 (2.0, 4.0)
Prior HCT		96 (30.9%)
Type of prior HCT	MUD	6 (6%)
	MRD	15 (16%)
	Haploidentical	5 (5%)
	Autologous	70 (73%)
CAR T-cell product	Axicabtagene ciloleucel	222 (71.4%)
	Tisagenlecleucel	25 (8.0%)
	Brexucabtagene autoleucel	19 (6.1%)
	Anti-CD19 trial product	43 (13.8%)
	Other	2 (0.6%)
Pre-CART LD chemotherapy	Fludarabine + Cyclophosphamide	296 (94.9%)
	Fludarabine + Bendamustine	5 (1.6%)
	Bendamustine Monotherapy	3 (1.0%)
…	Other	8 (2.6%)
ANC 30 D Pre-LD (×10^9^cells/L), median (IQR)		3.2 (2.0, 4.8)
ALC 30 D Pre-LD (×10^9^cells/L), median (IQR)		0.8 (0.5, 1.2)
ANC at LD Chemotherapy (×10^9^cells/L), median (IQR)		3.0 (2.0, 4.6)
ALC at LD chemotherapy (×10^9^cells/L) median (IQR)		0.6 (0.4, 1.0)
Hemoglobin at LD chemotherapy (g/dL), median (IQR)		10.4 (9.0, 11.8)
Platelets at LD chemotherapy (×10^9^cells/L), median (IQR)		163.5 (105.5, 217.5)
LDH at LD chemotherapy (IU/L), median (IQR)		240 (190.0, 314.0)
CRP at LD chemotherapy (mg/dL), median (IQR)		0.6 (0.2, 3.9)
Ferritin at LD chemotherapy (ng/mL), median (IQR)		485.8 (191.3, 1004.0)
CAR-HEMATOTOX score, median (IQR)		1.0 (1.0, 2.0)
CAR-HEMATOTOX score ≥ 2		133 (42.8%)
Relapse within 1 year of CART	Yes	124 (39.9%)
	No	145 (46.6%)
	Stable or progressive disease (never remitted)	40 (12.9%)
	Unknown	2 (0.6%)
30-D mortality		6 (1.9%)
1-Y mortality		80 (25.7%)
Cause of death	Relapse-related	59 (74%)
	Treatment-related	4 (5.0%)
	Infection-related	8 (10.0%)
	Other	2 (2.0%)
	Unknown	7 (9.0%)

IQR, interquartile range; CCI, Charlson Comorbidity Index; KPS, Karnofsky performance status; ECOG, Eastern Cooperative Oncology Group; DLBCL, diffuse large B-cell lymphoma; NHL, non-Hodgkin lymphoma; ALL, acute lymphocytic leukemia; CART, CAR T-cell therapy; CR, complete response; PR, partial response; VGPR, very good partial response; PD, progressive disease; SD, stable disease; HCT, hematopoietic cell transplant; MUD, matched unrelated donor; MRD, matched related donor; LD, lymphodepleting; ANC, absolute neutrophil count; ALC, absolute lymphocyte count; LDH, lactate dehydrogenase; CRP, C-reactive protein.

**Table 2.** Acute Toxicities and Complications Following CD19-directed Chimeric Antigen Receptor T-cell Therapy

Factor	Level	Value
*N*		311
Patients with CRS		261 (83.9%)
CRS Grade	1	109 (41.8%)
	2	144 (55.2%)
	3	8 (3.1%)
Received treatment for CRS		190 (72.8%)
First CRS therapy given	Steroid	5 (2.6%)
	Tocilizumab	85 (44.7%)
	Tocilizumab + Steroids	98 (51.6%)
	Siltuximab	1 (0.5%)
	Other	1 (0.5%)
Second CRS therapy given	Steroid	10 (17%)
	Tocilizumab	4 (7%)
	Tocilizumab + Steroids	39 (66%)
…	Anakinra	6 (10%)
Number of Tocilizumab doses given, median (IQR)		3.0 (2.0, 3.0)
Patients with ICANS		141 (45.3%)
ICANS Grade	1	18 (12.8%)
	2	62 (44.0%)
	3	48 (34.0%)
	4	10 (7.1%)
	Unknown	3 (2.1%)
Received treatment for ICANS		132 (93.6%)
ICANS treatment given	Steroid	122 (92.4%)
	Anakinra	6 (4.5%)
	Other^a^	4 (3.0%)
Febrile neutropenia following CART		185 (59.5%)
ICU admission following CART		45 (14.5%)

^a^Includes: steroids + anakinra (3) and steroid + rimiducid (1).

CRS, cytokine release syndrome; IQR, interquartile range; ICANS, immune effector cell-associated neurotoxicity syndrome; CART, CAR T-cell therapy; ICU, intensive care unit.

### Infections Within 1 Year of CART

**Figure 1.**
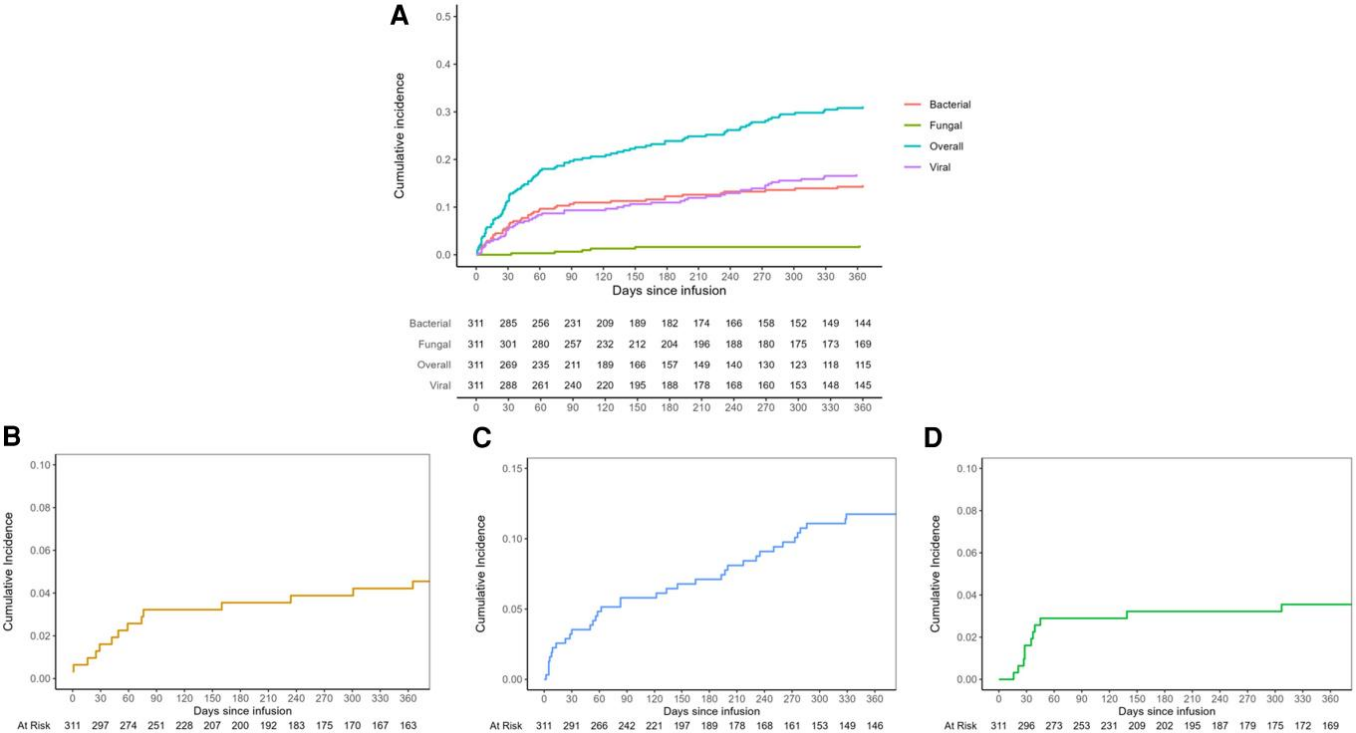
Cumulative incidence curves for postchimeric antigen receptor T-cell therapy (CART) infection by pathogen type (*A*), post-CART bloodstream infection (*B*), post-CART respiratory viral infection (*C*), post-CART cytomegalovirus infection (*D*).

Among 311 patients, 97 (31.2%) experienced at least one infection within a year of CART. In total, 131 infections were diagnosed (58 infections/100 person-years). By pathogen type, the cumulative incidence of infection was 19.0% (bacterial), 20.9% (viral), and 2.3% (fungal) (Figure 1*A*). No parasitic infections were diagnosed. The most common sites of infection were the upper respiratory tract and sinuses (18.3%), lower respiratory tract (16.8%), gastrointestinal tract (14.5%), genitourinary tract (14.5%), and bloodstream (13.0%). A majority of infections (62.0%) were Grade 1 (mild) in severity. Thirty-one (24.0%) were Grade 2 (moderate), and 13 (10.1%) were severe/life-threatening. Infection-related mortality was reported in eight patients (2.6%) in the first year. Of these, two deaths were due to infections after malignancy relapse, two were due to infectious syndromes with no identified etiology, one was related to septic shock in the setting of imaging evidence of acute cholecystitis, one was due to suspected secondary bacterial pneumonia following a SARS-CoV-2 infection, one was due to septic shock in the setting of grade 3 CMV infection complicated by candidemia, and the final death was related to progressive *Pneumocystis jirovecii* pneumonia (PJP).

Infection density was highest within the first 28 days following CART (161 infections/100 person-years) and diminished with time (Days 29–90: 84 infections/100 person-years; Days 91–365: 34 infections/100 person-years). Whereas there were similar rates of bacterial (Days 0–28: 50.0%; Days 29–90: 50.0%) and viral infections (Days 0–28: 47.4%; Days 29–90: 45.0%) early post-CART, viral infections predominated beyond Day 90 (54.7% viral vs 37.7% bacterial). Fungal infections were infrequent throughout the study period.

The median time to first infection was 49 days (IQR 21–165 days) with bacterial infections occurring earlier (median: 43 days, IQR 15–144 days) and viral (median: 60 days, IQR 28–236 days) and fungal infections (median: 100 days, IQR 33–150 days) occurring later post-CART.

### Microbiology of Post-CART Infections

The most frequently implicated bacterial pathogen was *Clostridioides difficile* (20.3%) followed by *Escherichia coli* (15.3%) and *Pseudomonas aeruginosa* (11.9%). The gastrointestinal tract (30.5%), genitourinary tract (30.5%), and bloodstream (27.1%) were the most common primary sites of bacterial infection (Table 3, Figure 1*B*). Of 12 *Clostridioides difficile* infections (CDI), 7 were diagnosed on the basis of a positive nucleic acid amplification test (NAAT) alone. A case each was diagnosed by a combination of positive NAAT and positive toxin enzyme immunoassay (EIA), positive NAAT, EIA, and glutamate dehydrogenase (GDH) assay, and positive GDH and toxin EIA. Two cases were confirmed by EIA alone.

**Table 3.** Details of Infectious Complications Occurring Within 1 Year of CD19-directed Chimeric Antigen Receptor T-Cell Therapy

Factor	Level	Value
*N*		131
Bacterial infection		59 (45.0%)
Bacterial pathogen	*Clostridioides difficile*	12 (20.3%)
	*Escherichia coli*	9 (15.3%)
	*Pseudomonas aeruginosa*	7 (11.9%)
	*Klebsiella pneumoniae*	5 (8.5%)
	*Campylobacter* species	3 (5.0%)
	*Enterobacter cloacae*	3 (5.0%)
	Coagulase-negative *Staphylococcus* species	2 (3.4%)
	*Haemophilus influenzae*	2 (3.4%)
	*Staphylococcus* species	2 (3.4%)
	*Achromobacter xylosoxidans/denitrificans*	1 (1.7%)
	*Citrobacter freundii*	1 (1.7%)
	*Klebsiella aerogenes*	1 (1.7%)
	*Enterococcus* species	1 (1.7%)
	*Enterococcus faecalis*	1 (1.7%)
	*Helicobacter pylori*	1 (1.7%)
	*Shigella species*	1 (1.7%)
	*Staphylococcus aureus*	1 (1.7%)
	*Streptococcus pneumoniae*	1 (1.7%)
	Viridans Group *Streptococcus*	1 (1.7%)
	*Yersinia enterocolitica*	1 (1.7%)
	Polymicrobial	3 (5.0%)
Site of bacterial infection	Gastrointestinal	18 (30.5%)
	Genitourinary	18 (30.5%)
	Bloodstream	16 (27.1%)
	Lower respiratory	3 (5.0%)
	Skin/soft tissue	3 (5.0%)
	Upper respiratory/sinus	1 (1.7%)
Bacterial resistance	ESBL Enterobacteriaceae	5 (8.5%)
	CRE	2 (3.4%)
	MDR Pseudomonas	1 (1.7%)
	MRSA	1 (1.7%)
	MRSE	1 (1.7%)
	None	49 (83.1%)
Fungal infection		7 (5.3%)
Fungal pathogen	*Pneumocystis jirovecii*	3 (43%)
	*Aspergillus* species	1 (14%)
	*Candida albicans*	1 (14%)
	*Candida glabrata*	2 (28%)
Site of fungal infection	Lower respiratory	4 (57%)
	Bloodstream only	1 (14%)
	Intraabdominal	1 (14%)
	Mucocutaneous	1 (14%)
Viral infection		65 (49.6%)
Viral pathogen	SARS-CoV-2	15 (23.1%)
	CMV	14 (21.5%)
	Rhinovirus	12 (18.5%)
	RSV	6 (9.2%)
	HSV	3 (4.6%)
	VZV	3 (4.6%)
	BK virus	2 (3.1%)
	HHV6	2 (3.1%)
	Non-SARS-CoV2 Coronavirus	2 (3.1%)
	Parainfluenza virus	2 (3.1%)
	Astrovirus	1 (1.5%)
	EBV	1 (1.5%)
	Unspecified Polyoma Virus	1 (1.5%)
	RSV + rhinovirus/enterovirus	1 (1.5%)
Site of viral infection	Upper respiratory/sinus	23 (35.4%)
	Disseminated	20 (30.8%)
	Lower respiratory	15 (23.1%)
	Skin/soft tissue	4 (6.2%)
	CNS	1 (1.5%)
	Gastrointestinal	1 (1.5%)
	Genitourinary	1 (1.5%)

MRSA, methicillin-resistant *Staphylococcus aureus*; ESBL, extended-spectrum beta-lactamase; CRE, carbapenem resistant Enterobacteriaceae; MDR, multidrug resistant; methicillin-resistant *Staphylococcus epidermidis*; CMV, Cytomegalovirus; SARS-CoV-2, Severe Acute Respiratory Syndrome Coronavirus 2; HHV6, Human Herpes Virus 6; RSV, Respiratory Syncytial Virus; VZV, Varicella Zoster Virus; EBV, Epstein-Barr Virus; HSV, Herpes Simplex Virus; CNS, central nervous system.

We reviewed patterns antimicrobial resistance in the bacterial isolates. Of 21 total *Enterobacteriaceae* isolates, 5 (23.8%) demonstrated an extended-spectrum beta-lactamase (ESBL) pattern of resistance. Two (5.4%) Gram-negative isolates were resistant to fluoroquinolones. Both were *Pseudomonas aeruginosa* bloodstream isolates that also displayed carbapenem resistance (Table 3).

The most frequently implicated viral pathogens were SARS-CoV-2 (23.0%) and CMV (22.0%). Clinically significant CMV infection tended to occur early after CART (median: 36 days, range 16–307 days), whereas SARS-CoV-2 infection occurred later (median: 235 days, range 58–329). Non-SARS-CoV-2 respiratory viruses accounted for most of the remaining viral infections (63.9%) (Table 3, Figure 1*C* and *D*).

Fungal infections were rare, with only seven events during the follow-up period. Two of these infections were fatal as previously described. In the fatal case of PJP, prophylaxis had not been prescribed for unclear reasons. Infection developed a month after a prednisone taper prescribed for pneumonitis. Two additional patients developed PJP, one of whom was on prophylaxis without other risk factors for infection. The second was diagnosed on day 362 in the setting of a steroid taper prescribed for sarcoidosis following completion of PJP prophylaxis. The only mold infection was a case of probable pulmonary *Aspergillus fumigatus* that developed in a patient prescribed a steroid taper for suspected pneumonitis. Additional details are provided in Supplementary Table 2.

### Characteristics and Outcomes based on Infection Status

We compared clinical characteristics and outcomes among patients who developed at least one infection within a year of CART and those who did not. While there was no difference in the proportion of patients with a prior HCT between groups, more patients in the infection group had undergone allogeneic HCT prior to CART (35% vs 24%, *P* = .049). There were no significant differences between groups in markers of baseline inflammation or hematopoietic reserve, but median baseline ferritin was nominally higher in the infection group (635.9 vs 394.0, *P* = .054). Similar proportions of patients developed CRS, however a larger proportion of patients who developed an infection had ICANS following CART (60.4% vs 38.6%, *P* < .001). Patients who developed infection were disproportionately more likely have been admitted to the intensive care unit during their CART admission (24.0% vs 10.2%, *P* = .001) (Table 4).

**Table 4.** Demographic, Clinical, and Treatment Characteristics of Patients Who did or did not Develop an Infectious Complication Within 1 Year of Chimeric Antigen Receptor T-cell Infusion

Factor	Level	No Infection	Infection	*P* Value
*N*		214	97	
Study site	Stanford	133 (62.1%)	62 (63.9%)	0.87
	UNC	36 (16.8%)	14 (14.4%)	
	Duke	45 (21.0%)	21 (21.6%)	
Age, median (IQR)		62.5 (52.0, 69.0)	63.0 (54.0, 71.0)	.62
Sex	Male	133 (62.1%)	62 (63.9%)	.84
	Female	81 (37.9%)	35 (36.1%)	
Race	African descent	13 (6.1%)	10 (10.3%)	.34
	Caucasian	151 (70.6%)	57 (58.8%)	
	Asian descent	19 (8.9%)	13 (13.4%)	
	Pacific Islander	2 (0.9%)	1 (1.0%)	
	Unknown	7 (3.3%)	2 (2.1%)	
	Other	22 (10.3%)	14 (14.4%)	
Ethnicity	Hispanic	22 (10.3%)	16 (16.5%)	.22
	Non-Hispanic	188 (87.9%)	78 (80.4%)	
	Unknown	4 (1.9%)	3 (3.1%)	
CCI, median (IQR)		4.0 (3.0, 5.0)	4.0 (3.0, 5.0)	.44
KPS	50	1 (0.5%)	1 (1.1%)	.94
	60	6 (2.9%)	4 (4.3%)	
	70	41 (20.1%)	20 (21.7%)	
	80	70 (34.3%)	29 (31.5%)	
	90	72 (35.3%)	30 (32.6%)	
	100	12 (5.9%)	6 (6.5%)	
	Unknown	2 (1.0%)	2 (2.2%)	
ECOG	0	47 (25.7%)	23 (31.9%)	.26
	1	111 (60.7%)	40 (55.6%)	
	2	23 (12.6%)	6 (8.3%)	
	3	0 (0.0%)	1 (1.4%)	
	Unknown	2 (1.1%)	2 (2.8%)	
Underlying malignancy	DLBCL	172 (80.4%)	69 (71.1%)	.26
	Non-DLBCL NHL	31 (14.5%)	21 (21.6%)	
	ALL	10 (4.7%)	7 (7.2%)	
	Other	1 (0.5%)	0 (0.0%)	
Disease status pre-CART	CR	3 (1.4%)	1 (1.0%)	.99
	PR	25 (11.7%)	12 (12.4%)	
	VGPR	2 (0.9%)	1 (1.0%)	
	PD	149 (69.6%)	65 (67.0%)	
	SD	34 (15.9%)	17 (17.5%)	
	Other	1 (0.5%)	1 (1.0%)	
Number of prior lines of chemotherapy, median (IQR)		3.0 (2.0, 4.0)	3.0 (2.0, 5.0)	.40
Prior HCT		70 (32.7%)	26 (26.8%)	.30
Type of prior HCT	MUD	4 (6.0%)	2 (8.0%)	.049
	MRD	12 (17.0%)	3 (12.0%)	
	Haploidentical	1 (1.0%)	4 (15.0%)	
	Autologous	53 (76.0%)	17 (65.0%)	
CAR T product	Axicabtagene ciloleucel	156 (72.9%)	66 (68.0%)	.55
	Tisagenlecleucel	18 (8.4%)	7 (7.2%)	
	Brexucabtagene autoleucel	10 (4.7%)	9 (9.3%)	
	Anti-CD19 trial product	29 (13.6%)	14 (14.4%)	
	Other	1 (0.5%)	1 (1.0%)	
Pre-CART LD chemotherapy	Fludarabine + Cyclophosphamide	202 (94.4%)	93 (95.9%)	.93
	Fludarabine + Bendamustine	4 (1.9%)	1 (1.0%)	
	Bendamustine monotherapy	2 (0.9%)	1 (1.0%)	
	Other	6 (2.8%)	2 (2.1%)	
ANC 30 D Pre-LD (×10^9^cells/L), median (IQR)		3.2 (2.0, 4.7)	3.0 (2.0, 5.1)	.78
ALC 30 D Pre-LD (×10^9^cells/L), median (IQR)		0.8 (0.5, 1.2)	0.8 (0.4, 1.2)	.86
ANC at LD chemotherapy (×10^9^cells/L), median (IQR)		2.9 (2.1, 4.4)	3.2 (2.0, 4.8)	.31
ALC at LD chemotherapy (×10^9^cells/L), median (IQR)		0.6 (0.4, 1.1)	0.6 (0.4, 1.0)	.55
Hemoglobin at LD chemotherapy (g/dL), median (IQR)		10.5 (9.2, 11.8)	10.3 (8.8, 11.4)	.25
Platelets at LD chemotherapy (×10^9^cells/L), median (IQR)		159.0 (106.0, 221.0)	165.0 (96.5, 210.0)	.72
LDH at LD chemotherapy (IU/L), median (IQR)		241.0 (191.0, 308.0)	234.0 (186.0, 350.0)	.63
CRP at LD chemotherapy (mg/dL), median (IQR)		0.6 (0.2, 3.9)	0.6 (0.2, 4.1)	.87
Ferritin at LD chemotherapy (ng/mL), median (IQR)		394.0 (179.0, 873.0)	635.9 (213.0, 1187.2)	.054
CAR-HEMATOTOX Score, median (IQR)		1.0 (1.0, 2.0)	1.0 (1.0, 3.0)	.35
CAR-HEMATOTOX Score ≥ 2		88 (41.1%)	45 (46.4%)	.38
Patients with CRS		178 (83.2%)	83 (85.6%)	.60
CRS Grade	1	76 (42.7%)	33 (39.8%)	.51
	2	98 (55.1%)	46 (55.4%)	
	3	4 (2.2%)	4 (4.8%)	
Received treatment for CRS		125 (70.2%)	65 (78.3%)	.17
First CRS therapy given	Steroid	3 (2.4%)	2 (3.1%)	.36
	Tocilizumab	50 (40.0%)	35 (53.8%)	
	Tocilizumab + Steroids	70 (56.0%)	28 (43.1%)	
	Siltuximab	1 (0.8%)	0 (0.0%)	
	Other	1 (0.8%)	0 (0.0%)	
Second CRS therapy given	Steroid	7 (17.0%)	3 (17.0%)	.74
	Tocilizumab	2 (5.0%)	2 (11.0%)	
	Tocilizumab + Steroids	27 (66.0%)	12 (67.0%)	
	Anakinra	5 (12.0%)	1 (6.0%)	
Number of Tocilizumab doses given, median (IQR)		3.0 (2.0, 3.0)	3.0 (2.0, 3.0)	.68
Patients with ICANS		82 (38.3%)	59 (60.8%)	<.001
ICANS Grade	1	12 (15.0%)	6 (10.0%)	.27
	2	39 (48.0%)	23 (39.0%)	
	3	27 (33.0%)	21 (36.0%)	
	4	3 (4.0%)	7 (12.0%)	
	Unknown	1 (1.0%)	2 (3.0%)	
Received treatment for ICANS		76 (93.0%)	56 (95.0%)	.59
ICANS treatment given	Steroid	70 (92.0%)	52 (93.0%)	.73
	Anakinra	3 (4.0%)	3 (5.0%)	
	Other	3 (4.0%)	1 (2.0%)	
Febrile neutropenia following CART		120 (56.1%)	65 (67.0%)	.069
ICU admission following CART		22 (10.3%)	23 (23.7%)	.002
30-D mortality		4 (1.9%)	2 (2.1%)	.91
1-Y mortality		56 (26.2%)	24 (24.7%)	.79

IQR, interquartile range; CCI, Charlson Comorbidity Index; KPS, Karnofsky performance status; ECOG, Eastern Cooperative Oncology Group; DLBCL, diffuse large B-cell lymphoma; NHL, non-Hodgkin lymphoma; ALL, acute lymphocytic leukemia; CAR, chimeric antigen receptor; CR, complete response; PR, partial response; VGPR, very good partial response; PD, progressive disease; SD, stable disease; HCT, hematopoietic cell transplant; MUD, matched unrelated donor; MRD, matched related donor; LD, lymphodepleting; ANC, absolute neutrophil count; ALC, absolute lymphocyte count; LDH, lactate dehydrogenase; CRP, C-reactive protein; CRS, cytokine release syndrome; ICANS, immune effector cell-associated neurotoxicity syndrome; ICU, intensive care unit.

There was no difference in the proportion of patients who relapsed within the first year following CART based on infection status. Thirty-day mortality was low in both groups and 1-year mortality rates were similar (Table 4).

### Time-to-Event and Logistic Regression Analyses

We performed a time-to-event analysis to assess for independent predictors of post-CART infection using Fine-Gray subdistribution hazard models. Although prior haploidentical HCT, baseline ferritin, CAR HEMATOTOX score, ICANS, and duration of fluoroquinolone therapy were associated with elevated hazard of infection in univariate analysis, none of these associations remained significant in an age- and underlying disease-adjusted multivariate model (Supplementary Table 3).

We performed exploratory logistic regression analyses to assess for predictors of infection >90 days post-CART, ≤90 days post-CART, post-CART bacterial infection, and post-CART viral infection. While on univariate we did observe associations between markers of baseline inflammatory state (ferritin), baseline hematopoietic reserve (hemoglobin, CAR HEMATOTOX score, neutropenia, and lymphopenia), antibiotic exposure, and the outcomes of interest, only an association between baseline neutropenia and viral infection (HR 1.94, 95% CI 1.02, 3.68) remained significant on multivariate analysis (Supplementary Tables 4–8).

## DISCUSSION

Our understanding of post-CART infections is largely based on data from single-center studies. Here, we endeavored to add meaningfully to the literature by performing a multicenter study examining the epidemiology of and risk factors for post-CART infections. We found that infections occurred in nearly a third of patients within a year of CART, and a majority were mild or moderate in severity. Though we were not able to identify independent predictors of infection, the epidemiology of post-CART infections has important implications for prevention and management of these complications.

Among 311 patients in our cohort, 31.2% developed at least one infection in the first year post-CART. Infection density was highest in the first month post-CART and diminished with time. As in past studies, incidence of bacterial infections was higher than that of viral infections in the first month post-CART while viral infections predominated after Day 90 [6, 10, 13, 15, 18, 19, 24]. Most infections were mild in severity (62.0%) and infection-related mortality was reported in 2.6% of patients. Comparing these measures of infection incidence to those in the existing literature is difficult due to a lack of standardized definitions for infection and varied reporting methods across studies. Infection rates between 10% and 80% have been reported, with studies requiring microbiologic confirmation of infection reporting lower rates [10, 25]. The infection rate in our cohort is lower than most studies with similar follow-up time, but all of these include both clinically and microbiologically diagnosed infections [6, 8, 9, 16–18, 26]. Highlighting these differences is important given the highly subjective nature of clinical diagnoses, the large clinical overlap between infection and acute CART toxicities, and the impact that misclassification of these events may have on risk factor analyses.

Viral infections had the highest cumulative incidence in our cohort (20.9%) followed by bacterial (19.0%) and fungal infections (2.3%). The most commonly implicated bacterial pathogen was *C. difficile*. Although CDI is commonly reported in studies of post-CART infection, this finding should be interpreted with caution [6, 8, 13–15, 18, 24, 25]. Seven of 12 cases in our cohort were diagnosed by positive NAAT alone, suggesting that colonization may frequently be misclassified as infection. Infection due to Gram-negative bacilli was also common and it should be noted that 23.8% of *Enterobacteriaceae* isolates demonstrated resistance patterns consistent with ESBL production, a finding that may have important implications for prophylactic and empiric antimicrobial decisions.

Respiratory viruses caused the majority of viral infections in our cohort, but 30 of 36 were graded as mild in severity. Although SARS-CoV-2 has been associated with poor outcomes in CART recipients, it is notable that only one case in our cohort was severe [27]. These infections occurred late, allowing more time for immune reconstitution, which may have impacted their severity. CMV reactivation following CART is receiving growing attention in the literature. While the rate of CMV reactivation was beyond the scope of this study, the rate of treated CMV viremia events was similar to other published reports [28]. These infections tended to occur early post-CART and were characterized by viremia without end-organ disease. None of the study sites followed a standardized CMV surveillance or treatment initiation protocol, limiting our ability to comment on optimal post-CART CMV management.

Fungal infections were rare, but with two fatal outcomes, represent clinically significant post-CART complications. The use of fluconazole prophylaxis during neutropenia varies by institution and although three cases of invasive candidiasis occurred at a site that does not use prophylaxis, the infections did not occur during an episode of neutropenia [12]. PJP prophylaxis is more universally applied post-CART and the incidence of 3 cases of infections in our cohort—one of them fatal—lends support to this practice, though the appropriate duration deserves further study.

Although our risk factor analysis did not identify independent predictors of post-CART infection, the associations identified on univariate analysis map to important domains of risk established by past studies [8, 10, 13, 14, 17, 19, 25]. These include markers of hematopoietic reserve or baseline inflammatory state and acute toxicities and their management. As far as we are aware, ours is the first study to include antimicrobial exposure in a risk factor analysis. Although the associations were not significant on the adjusted analysis, the univariate associations between antimicrobial duration and infection points to a potential third risk domain that warrants attention in future work.

Our study has several limitations. Although the inclusion of three sites afforded a larger sample size, this is still a retrospective study subject to unmeasured confounding. The retrospective design also limited our ability to include potentially important markers of immune reconstitution, such as immunoglobulin levels and CD4+ T-cell counts in our risk factor analysis since they were inconsistently measured across the cohort resulting in a high degree of missingness. Many of the variables in our dataset were manually abstracted from medical records, and their accuracy may have been impacted by incomplete reporting. In particular, it is possible that infections diagnosed or antibiotics prescribed at outside centers were not captured. Further, with few exceptions, we elected to include only microbiologically confirmed infections. Although more specific, these definitions may have resulted in misclassification of infections that are seldom confirmed microbiologically, such as respiratory tract infections. CART recipients are particularly susceptible to respiratory tract infections, but they are likely underreported in this study.

Our study also has limitations affecting its generalizability. A majority of patients in the cohort received axicabtagene ciloleucel. The results may therefore not be generalizable to recipients of other CD19-directed CAR T-cell products. In addition, our study spanned the early part of the SARS-CoV-2 pandemic. Healthcare and community mitigation strategies may have impacted the epidemiology of infections in our cohort both directly by altering patterns of infection transmission and indirectly by affecting patient access to diagnostic testing. The external validity of these results may have been impacted by the unique clinical context under which the data were collected.

This study contributes to the evolving understanding of post-CART infection. Infections are common, occurring in close to a third of patients within a year of CART, yet are often mild in severity. Bacterial bloodstream, urinary tract, and *C. difficile* infections were common early after CART, but respiratory viral infections predominated beyond 90 days post-CART. Fungal infections are rare, but clinically meaningful complications in this population. Though we were not able to identify independent predictors of post-CART infection, elucidation of their epidemiology may help further optimize care of this vulnerable patient group.

## Supplementary Material

ofag423_Supplementary_Data

## Supplementary Data

Supplementary materials are available at *Open Forum Infectious Diseases* online. Consisting of data provided by the authors to benefit the reader, the posted materials are not copyedited and are the sole responsibility of the authors, so questions or comments should be addressed to the corresponding author.
