# Supplementary material for: Infectious Complications of CD19-targeted Chimeric Antigen Receptor T-cell Therapy: a Multicenter Cohort Study
Source: Open Forum Infect Dis. 2026 Jul 3;13(7):ofag423. doi: 10.1093/ofid/ofag423 (PMC13371758; doi:10.1093/ofid/ofag423)
Supplement: ofag423_Supplementary_Data [file ofag423_supplementary_data.docx]

SUPPLEMENTARY TABLES

|  | Stanford University | Duke University | University of North Carolina – Chapel Hill |
| --- | --- | --- | --- |
| Viral Prophylaxis | Acyclovir 800mg twice daily (starting at day +1 for 18-24 months) | Acyclovir 400mg twice daily (starting with LD chemotherapy for ≥6 months and until CD4 > 200 cells/uL) | Valacyclovir 500mg twice daily (starting day 0 for ≥12 months an until CD4 > 200 cells/uL) |
| Bacterial Prophylaxis | Levofloxacin 750mg daily (while ANC <500 cells/uL) | Levofloxacin 500mg daily (starting with LD chemotherapy until ANC >1000 cells/uL) | Levofloxacin 500mg daily (while ANC <500 cells/uL)* |
| Fungal Prophylaxis | None | Fluconazole 400mg daily (starting with LD chemotherapy until ANC >1000 cells/uL) | No history of invasive mold infection: Fluconazole 400mg daily (while ANC <500 cells/uL) |
|  |  |  | History of invasive mold infection: Posaconazole 300mg daily or prior antifungal (while ANC <500 cells/uL) |
| PJP Prophylaxis | Atovaquone 1500mg daily (starting on day of discharge, or day +14 if outpatient, for 18-24 months)** | TMP-SMX 1 DS tablet thrice weekly (starting with LD chemotherapy for ≥6 months and until CD4 > 200 cells/uL) | TMP-SMX 1 DS tablet thrice weekly (starting day 0 for ≥6 months and until CD4 > 200 cells/uL) |

**Table S1.** Anti-infective prophylaxis for CD19-directed chimeric antigen receptor T-cell recipients by study institution. *Cefdinir 300mg daily may be used in penicillin-allergic patients. **May consider transition to trimethoprim-sulfamethoxazole if cell counts permit. LD, lymphodepleting; ANC, absolute neutrophil count; PJP, *Pneumocystis jirovecii* pneumonia; TMP-SMX, trimethoprim-sulfamethoxazole; DS, double strength

|  | Day of Infection (post-CART) | Pathogen | Proven/Probable | Site | Prophylaxis at Diagnosis | Infection Grade  (COD Y/N) | Case Details |
| --- | --- | --- | --- | --- | --- | --- | --- |
| 1 | 33 | *Candida albicans* | Proven | Blood | None | 3 (Y) | - Non-neutropenic  - CVC in place  - No TPN  - No relevant comorbidities  - No intraabdominal source on imaging |
| 2 | 27 | *Candida glabrata* | Proven | Mucocutaneous | None | 1 (N) | - Necrotic tonsillar mass with exudate on exam  - Tissue was sampled and cultrures grew *C. glabrata* |
| 3 | 74 | *Candida glabrata* | Proven | Intraabdominal | None | 3 (N) | - Long-standing enteroenteric/enterocolonic fistula complicated by acute abscess  - Drain placed with abscess cultures growing *C. glabrata* |
| 4 | 108 | *Pneumocystis jirovecii* | Proven | Lower Respiratory Tract | None | 3 (Y) | - Prescribed prednisone taper starting at 1mg/kg/day one month prior for pneumonitis  - Not prescribed PJP prophylaxis for unclear reasons  - Progressed to respiratory failure requiring mechanical ventilation and death |
| 5 | 100 | *Pneumocystis jirovecii* | Proven | Lower Respiratory Tract | TMP-SMX | 2 (N) | - Not prescribed antecedent steroids  - Developed hypoxia requiring high-flow nasal cannula |
| 6 | 362 | *Pneumocystis jirovecii* | Proven | Lower Respiratory Tract | None | 2 (N) | - Completed pentamidine prophylaxis  - Steroid taper for presumed pulmonary sarcoidosis  - Developed hypoxia requiring up to 5L supplementary oxygen |
| 7 | 150 | *Aspergillus fumigatus* | Probable | Lower Respiratory Tract | None | 2 (N) | - Pulmonary consolidation consistent with IPA  - Sputum culture grew *A. fumigatus*  - Non-neutropenic  - Prescribed 15 day prednisone taper starting at 40mg daily for pneumonitis immediately prior  - Successfully treated with posaconazole |

**Table S2.** Details of Fungal Infections. CART, chimeric antigen receptor T-cell therapy; COD, cause of death; CVC, central venous catheter; TPN, total parenteral neutrition; PJP, *Pneumocystis jirovecii;* TMP-SMX, trimethoprim-sulfamethoxazole; IPA, invasive pulmonary aspergillosis.

|  |  | Univariate Analysis | | | Multivariate analysis | | |
| --- | --- | --- | --- | --- | --- | --- | --- |
| Variable |  | HR | 95% C.I. | p | HR | 95% C.I. | p |
| Age |  | 1.00 | (0.98, 1.01) | 0.74 | 1.00 | (0.98, 1.02) | 0.93 |
| Sex |  |  |  |  |  |  |  |
|  | Male | Reference |  |  |  |  |  |
|  | Female | 0.96 | (0.63, 1.45) | 0.83 |  |  |  |
| Race |  |  |  |  |  |  |  |
|  | Caucasian | Reference |  |  |  |  |  |
|  | African descent | 1.73 | (0.90, 3.31) | 0.10 |  |  |  |
|  | Asian descent | 1.45 | (0.81, 2.59) | 0.21 |  |  |  |
|  | Pacific Islander | 1.26 | (0.17, 9.36) | 0.82 |  |  |  |
|  | Other | 1.61 | (0.88, 2.93) | 0.12 |  |  |  |
|  | Unknown | 0.72 | (0.20, 2.58) | 0.61 |  |  |  |
| Patient ethnicity |  |  |  |  |  |  |  |
|  | Non-Hispanic | Reference |  |  |  |  |  |
|  | Hispanic | 1.66 | (0.96, 2.86) | 0.070 |  |  |  |
|  | Unknown | 1.18 | (0.44, 3.17) | 0.75 |  |  |  |
| Study site |  |  |  |  |  |  |  |
|  | Duke | Reference |  |  |  |  |  |
|  | Stanford | 1.01 | (0.62, 1.67) | 0.96 |  |  |  |
|  | UNC | 0.92 | (0.46, 1.81) | 0.80 |  |  |  |
| CCI |  | 0.97 | (0.90, 1.05) | 0.44 |  |  |  |
| KPS |  | 1.00 | (0.98, 1.02) | 0.67 |  |  |  |
| ECOG |  | 1.01 | (0.99, 1.02) | 0.35 |  |  |  |
| Number of Prior Lines of Chemotherapy |  | 1.06 | (1.16, 0.96) | 0.25 |  |  |  |
| Disease status Pre-CTTx |  |  |  |  |  |  |  |
|  | CR | Reference |  |  |  |  |  |
|  | PD | 1.73 | (0.29, 10.39) | 0.55 |  |  |  |
|  | PR | 1.64 | (0.25, 10.61) | 0.60 |  |  |  |
|  | SD | 1.92 | (0.30, 12.13) | 0.49 |  |  |  |
|  | Other | 4.14 | (0.65, 26.27) | 0.13 |  |  |  |
| Underlying Malignancy |  |  |  |  |  |  |  |
|  | ALL | Reference |  |  | Reference |  |  |
|  | DLBCL | 0.52 | (0.24, 1.12) | 0.094 | 0.55 | (0.20, 1.50) | 0.25 |
|  | non-DLBCL NHL | 0.70 | (0.30, 1.60) | 0.39 | 0.68 | (0.25, 1.88) | 0.46 |
| Prior HCT |  | 0.80 | (0.51, 1.25) | 0.33 |  |  |  |
| Type of Prior HCT |  |  |  |  |  |  |  |
|  | Autologous |  |  |  |  |  |  |
|  | Haploidentical | 4.19 | (1.78, 9.89) | 0.0011 |  |  |  |
|  | MRD | 0.98 | (0.28, 3.44) | 0.98 |  |  |  |
|  | MUD | 2.17 | (0.54, 8.68) | 0.27 |  |  |  |
| CAR T-cell product |  |  |  |  |  |  |  |
|  | Anti-CD19 trial product | Reference |  |  |  |  |  |
|  | Tisagenlecleucel | 0.96 | (0.39, 2.35) | 0.92 |  |  |  |
|  | Brexucabtagene autoleucel | 1.42 | (0.62, 3.25) | 0.40 |  |  |  |
|  | Axicabtagene ciloleucel | 0.81 | (0.46, 1.41) | 0.45 |  |  |  |
|  | Other | 1.18 | (0.21, 6.52) | 0.85 |  |  |  |
| Pre-CTTx LD Chemotherapy |  |  |  |  |  |  |  |
|  | Bendamustine | Reference |  |  |  |  |  |
|  | Fludarabine/ Bendamustine | 0.70 | (0.06, 8.61) | 0.78 |  |  |  |
|  | Fludarabine/ Cyclophosphamide | 1.01 | (0.21, 4.93) | 1.00 |  |  |  |
|  | Other | 1.00 | (0.13, 7.45) | 1.00 |  |  |  |
| ANC 30 Days Pre-LD |  | 0.96 | (0.90, 1.02) | 0.14 |  |  |  |
| ALC 30 Days Pre-LD |  | 0.96 | (0.90, 1.01) | 0.14 |  |  |  |
| ANC at LD Chemotherapy |  | 1.07 | (0.99, 1.15) | 0.074 |  |  |  |
| ALC at LD Chemotherapy |  | 0.86 | (0.64, 1.15) | 0.30 |  |  |  |
| Hemoglobin at LD Chemotherapy |  | 0.90 | (0.81, 1.01) | 0.07 |  |  |  |
| Platelets at LD Chemotherapy |  | 1.00 | (1.00, 1.00) | 0.71 |  |  |  |
| CRP at LD Chemotherapy |  | 1.03 | (0.95, 1.12) | 0.45 |  |  |  |
| Ferritin at LD Chemotherapy |  | 1.17 | (1.02, 1.34) | 0.030 |  |  |  |
| LDH at LD chemotherapy |  | 1.27 | (0.96, 1.68) | 0.088 |  |  |  |
| CAR-HEMATOX Score |  | 1.17 | (1.02, 1.34) | 0.030 | 1.10 | (0.94, 1.28) | 0.21 |
| Neutropenic at Day 0 |  | 1.54 | (0.97, 2.45) | 0.07 |  |  |  |
| Lymphopenic at Day 0 |  | 1.17 | (0.43, 3.16) | 0.76 |  |  |  |
| CRS after CAR T-cell infusion |  | 1.12 | (0.65, 1.94) | 0.69 |  |  |  |
| Grade of CRS |  | 1.22 | (0.80, 1.85) | 0.35 |  |  |  |
| Received Treatment for CRS |  | 1.43 | (0.85, 2.38) | 0.18 |  |  |  |
| First CRS Therapy Given |  |  |  |  |  |  |  |
|  | Tocilizumab | Reference |  |  |  |  |  |
|  | Tocilizumab + Steroids | 0.63 | (0.39, 1.03) | 0.068 |  |  |  |
|  | Other | 0.54 | (0.14, 2.17) | 0.39 |  |  |  |
| Number Tocilizumab Doses Given |  | 1.02 | (0.81, 1.28) | 0.87 |  |  |  |
| ICANS after CAR T-cell infusion |  | 1.56 | (1.05, 2.32) | 0.030 | 1.48 | (0.97, 2.27) | 0.070 |
| Grade of ICANS |  | 1.02 | (1.01, 1.03) | 0.0032 |  |  |  |
| Treated for ICANS |  | 1.48 | (0.32, 6.85) | 0.613 |  |  |  |
| ICANS Treatment Given |  |  |  |  |  |  |  |
|  | Steroid | Reference |  |  |  |  |  |
|  | Tocilizumab + Steroids | 0.88 | (0.48, 1.61) | 0.68 |  |  |  |
|  | Other | 0.90 | (0.32, 2.54) | 0.84 |  |  |  |
| Febrile Neutropenia Following CTTx |  | 1.41 | (0.93, 2.13) | 0.11 |  |  |  |
| Immunosuppressive exposure |  | 1.17 | (0.79, 1.75) | 0.44 |  |  |  |
| Immunosuppressive Therapy Duration |  | 1.02 | (1.00, 1.04) | 0.094 |  |  |  |
| Fluoroquinolone Duration |  | 1.03 | (1.00, 1.05) | 0.020 | 1.01 | (0.99, 1.04) | 0.31 |
| Vancomycin Duration |  | 0.99 | (0.95, 1.03) | 0.46 |  |  |  |
| Carbapenem Duration |  | 1.02 | (0.99, 1.05) | 0.26 |  |  |  |
| Beta-lactam Duration |  | 0.99 | (0.98, 1.01) | 0.32 |  |  |  |

**Table S3**. Univariate and multivariate Fine-Gray subdistribution hazard models to evaluate association between baseline clinical characteristics and development of infection within 1 year of chimeric antigen receptor T-cell therapy (CTTx) with malignancy relapse or death as a competing risks. CCI, Charlson Comorbidity Index; KPS, Karnofsky Performance Status; ECOG, Eastern Cooperative Oncology Group; CTTx, chimeric antigen receptor T-cell therapy; CR, Complete Response; PD, Progressive Disease; PR, Partial Response; SD, Stable Disease; ALL, Acute Lymphocytic Leukemia; DLBCL, Diffuse Large B-cell Lymphoma; NHL, Non-Hodgkin Lymphoma; HCT, Hematopoietic Cell Transplant; MUD, Matched Unrelated Donor; MRD, Matched Related Donor; CAR, Chimeric Antigen Receptor; LD, Lymphodepleting; ANC, Absolute Neutrophil Count; ALC, Absolute Lymphocyte Count; CRP, C-Reactive Protein; LDH, Lactate Dehydrogenase; CRS, cytokine release syndrome; ICANS, Immune Effector Cell-Associated Neurotoxicity Syndrome

| Variable |  | OR | 95% C.I. | p |
| --- | --- | --- | --- | --- |
| Age |  | 0.99 | (0.97, 1.01) | 0.22 |
| Sex |  |  |  |  |
|  | Male | Reference |  |  |
|  | Female | 1.23 | (0.73, 2.09) | 0.43 |
| Patient Race |  |  |  |  |
|  | Caucasian | Reference |  |  |
|  | African descent | 1.28 | (0.48, 3.40) | 0.96 |
|  | Asian descent | 1.65 | (0.73, 3.73) | 0.95 |
|  | Pacific Islander | -- | -- | -- |
|  | Other | 2.05 | (0.96, 4.36) | 0.95 |
|  | Unknown | 2.90 | (0.75, 11.24) | 0.94 |
| Patient ethnicity |  |  |  |  |
|  | Non-Hispanic | Reference |  |  |
|  | Hispanic | 1.71 | (0.83, 3.54) | 0.87 |
|  | Unknown | 2.47 | (0.54, 11.33) | 0.42 |
| Study site |  |  |  |  |
|  | Duke | Reference |  |  |
|  | Stanford | 0.87 | (0.46, 1.64) | 0.85 |
|  | UNC | 0.84 | (0.36, 1.96) | 0.78 |
| CCI |  | 0.95 | (0.83, 1.09) | 0.45 |
| KPS |  | 1.00 | (0.97, 1.03) | 0.96 |
| ECOG |  | 1.00 | (0.98, 1.02) | 0.97 |
| Number of prior lines of chemotherapy |  | 0.96 | (0.83, 1.11) | 0.58 |
| Disease status Pre-CART |  |  |  |  |
|  | CR | Reference |  |  |
|  | PD | 0.30 | (0.04, 2.16) | 0.98 |
|  | PR | 0.44 | (0.06, 3.54) | 0.98 |
|  | SD | 0.37 | (0.05, 2.92) | 0.98 |
|  | Other | -- | -- | -- |
| Underlying Malignancy |  |  |  |  |
|  | ALL |  |  |  |
|  | DLBCL | 0.47 | (0.17, 1.28) | 0.982 |
|  | non-DLBCL NHL | 0.37 | (0.12, 1.21) | 0.98 |
| Prior HCT |  | 1.24 | (0.72, 2.14) | 0.43 |
| Type of Prior HCT |  |  |  |  |
|  | Autologous | Reference |  |  |
|  | Haploidentical | 5.06 | (0.78, 32.98) | 0.29 |
|  | MRD | 1.69 | (0.50, 5.66) | 0.54 |
|  | MUD | 3.38 | (0.62, 18.38) | 0.58 |
| CAR T-cell product |  |  |  |  |
|  | Anti-CD19 trial |  |  |  |
|  | Tisagenlecleucel | 1.45 | (0.51, 4.17) | 0.52 |
|  | Brexucabtagene autoleucel | 0.48 | (0.12, 1.97) | 0.17 |
|  | Axicabtagene ciloleucel | 0.81 | (0.39, 1.69) | 0.41 |
|  | Other | 2.58 | (0.15, 44.70) | 0.45 |
| Lymphodepleting (LD) chemotherapy |  |  |  |  |
|  | Bendamustine | Reference |  |  |
|  | Fludarabine/ Bendamustine | -- | -- | -- |
|  | Fludarabine/ Cyclophosphamide | 0.65 | (0.06, 7.23) | 0.99 |
|  | Other | 3.33 | (0.20, 54.53) | 0.98 |
| ANC 30 Days Pre-LD |  | 0.94 | (0.87, 1.01) | 0.10 |
| ALC 30 Days Pre-LD |  | 0.95 | (0.88, 1.02) | 0.16 |
| ANC at LD Chemotherapy |  | 1.01 | (0.92, 1.10) | 0.86 |
| ALC at LD Chemotherapy |  | 0.96 | (0.72, 1.26) | 0.74 |
| Hemoglobin at LD Chemotherapy |  | 1.02 | (0.89, 1.18) | 0.76 |
| Platelets at LD Chemotherapy |  | 1.00 | (1.00, 1.00) | 0.63 |
| CRP at LD Chemotherapy |  | 0.98 | (0.96, 1.01) | 0.24 |
| Ferritin at LD Chemotherapy |  | 1.07 | (0.91, 1.25) | 0.43 |
| LDH at LD chemotherapy |  | 1.13 | (0.78, 1.64) | 0.53 |
| CAR-HEMATOX Score |  | 1.06 | (0.89, 1.27) | 0.51 |
| CRS after CAR T-cell infusion |  | 0.57 | (0.30, 1.10) | 0.09 |
| Grade of CRS |  | 0.92 | (0.54, 1.56) | 0.76 |
| Received Treatment for CRS |  | 1.38 | (0.71, 2.70) | 0.35 |
| First CRS Therapy Given |  |  |  |  |
|  | Tocilizumab Only | Reference |  |  |
|  | Tocilizumab + Steroids | 0.51 | (0.26, 1.00) | 0.96 |
|  | Other | -- | -- | -- |
| Number Tocilizumab Doses Given |  | 1.05 | (0.75, 1.45) | 0.79 |
| ICANS after CAR T-cell infusion |  | 1.47 | (0.88, 2.47) | 0.14 |
| Grade of ICANS |  | 1.00 | (0.98, 1.03) | 0.81 |
| Treated for ICANS |  | -- | -- | -- |
| ICANS Treatment Given |  |  |  |  |
|  | Steroid Only | Reference |  |  |
|  | Tocilizumab + Steroids | 1.46 | (0.65, 3.29) | 0.23 |
|  | Other | 0.60 | (0.12, 3.03) | 0.39 |
| Febrile Neutropenia Following CART |  | 0.94 | (0.56, 1.60) | 0.83 |
| Neutropenic at Day 0 |  | 1.08 | (0.57, 2.04) | 0.82 |
| Neutropenic at Day 30 |  | 1.39 | (0.65, 2.99) | 0.40 |
| Neutropenic at Day 60 |  | 3.43 | (1.34, 8.79) | 0.010 |
| Neutropenic at Day 90 |  | 1.15 | (0.39, 3.37) | 0.80 |
| Lymphopenic at Day 0 |  | 0.92 | (0.28, 2.97) | 0.88 |
| Lymphopenic at Day 30 |  | 1.62 | (0.78, 3.34) | 0.19 |
| Lymphopenic at Day 60 |  | 1.69 | (0.64, 4.45) | 0.29 |
| Lymphopenic at Day 90 |  | 1.04 | (0.36, 3.01) | 0.95 |
| Immunosuppressive exposure |  | 1.26 | (0.75, 2.11) | 0.38 |
| Immunosuppressive Therapy Duration |  | 1.00 | (0.97, 1.04) | 0.86 |
| Fluoroquinolone Duration |  | 1.01 | (0.99, 1.04) | 0.16 |
| Vancomycin Duration |  | 1.04 | (1.01, 1.06) | 0.011 |
| Carbapenem Duration |  | 1.03 | (1.00, 1.06) | 0.10 |
| Beta-lactam Duration |  | 1.01 | (1.00, 1.03) | 0.031 |

**Table S4.** Univariate logistic regression to evaluate for association between exposures of interest and risk of infection after day +90 following chimeric antigen receptor T-cell infusion. CCI, Charlson Comorbidity Index; KPS, Karnofsky Performance Status; ECOG, Eastern Cooperative Oncology Group; CART, chimeric antigen receptor T-cell therapy; CR, Complete Response; PD, Progressive Disease; PR, Partial Response; SD, Stable Disease; ALL, Acute Lymphocytic Leukemia; DLBCL, Diffuse Large B-cell Lymphoma; NHL, Non-Hodgkin Lymphoma; HCT, Hematopoietic Cell Transplant; MUD, Matched Unrelated Donor; MRD, Matched Related Donor; CAR, Chimeric Antigen Receptor; LD, Lymphodepleting; ANC, Absolute Neutrophil Count; ALC, Absolute Lymphocyte Count; CRP, C-Reactive Protein; LDH, Lactate Dehydrogenase; CRS, cytokine release syndrome; ICANS, Immune Effector Cell-Associated Neurotoxicity Syndrome

| Variable |  | OR | UCL | P |
| --- | --- | --- | --- | --- |
| Age |  | 0.99 | (0.97, 1.01) | 0.99 |
| Sex |  |  |  |  |
|  | Male | Reference |  |  |
| Female | Female | 1.19 | (0.68, 2.09) | 0.54 |
| Race |  |  |  |  |
|  | Caucasian | Reference |  |  |
|  | African descent | 2.02 | (0.78, 5.26) | 0.95 |
|  | Asian descent | 1.29 | (0.52, 3.22) | 0.96 |
|  | Pacific Islander | 2.31 | (0.20, 26.16) | 0.94 |
|  | Other | 2.31 | (1.06, 5.04) | 0.94 |
|  | Unknown | -- | -- | -- |
| Ethnicity |  |  |  |  |
|  | Non-Hispanic | Reference |  |  |
|  | Hispanic | 2.25 | (1.08, 4.70) | 0.13 |
|  | Unknown | 0.72 | (0.09, 6.12) | 0.50 |
| Study site |  |  |  |  |
|  | Duke |  |  |  |
|  | Stanford | 0.99 | (0.50, 1.96) | 0.76 |
|  | UNC | 0.82 | (0.32, 2.07) | 0.63 |
| CCI |  | 0.94 | (0.80, 1.09) | 0.39 |
| KPS |  | 0.99 | (0.96, 1.02) | 0.38 |
| ECOG |  | 1.02 | (1.00, 1.04) | 0.13 |
| Number of prior lines of chemotherapy |  | 1.11 | (0.96, 1.27) | 0.17 |
| Disease status Pre-CART |  |  |  |  |
|  | Non-Responder (PD or SD) | Reference |  |  |
|  | Responder (PR or CR) | 0.84 | (0.37, 1.90) | 0.67 |
| Underlying Malignancy |  |  |  |  |
|  | ALL | Reference |  |  |
|  | DLBCL | 0.45 | (0.16, 1.27) | 0.99 |
|  | non-DLBCL NHL | 0.48 | (0.15, 1.59) | 0.99 |
| Prior HCT |  | 0.84 | (0.46, 1.54) | 0.57 |
| Type of Prior HCT |  |  |  |  |
|  | Autologous | Reference |  |  |
|  | Haploidentical | 2.92 | (0.44, 19.31) | 0.22 |
|  | MRD | 0.68 | (0.14, 3.36) | 0.43 |
|  | MUD | 0.88 | (0.09, 8.16) | 0.76 |
| CAR T-cell product |  |  |  |  |
|  | Anti-CD19 trial | Reference |  |  |
|  | Tisagenlecleucel | 1.04 | (0.33, 3.32) | 0.97 |
|  | Brexucabtagene autoleucel | 1.52 | (0.46, 5.05) | 0.96 |
|  | Axicabtagene ciloleucel | 0.77 | (0.35, 1.69) | 0.97 |
|  | Other | -- | -- | -- |
| Lymphodepleting (LD) chemotherapy |  |  |  |  |
|  | Bendamustine-containing Regimen | Reference |  |  |
|  | Fludarabine/ Cyclophosphamide | 1.86 | (0.23, 15.42) | 0.42 |
|  | Other | 1.00 | (0.05, 19.36) | 0.80 |
| ANC 30 Days Pre-LD |  | 0.97 | (0.90, 1.05) | 0.47 |
| ALC 30 Days Pre-LD |  | 0.97 | (0.90, 1.05) | 0.45 |
| ANC at LD Chemotherapy |  | 1.07 | (0.98, 1.17) | 0.14 |
| ALC at LD Chemotherapy |  | 0.94 | (0.64, 1.39) | 0.76 |
| Hemoglobin at LD Chemotherapy |  | 0.84 | (0.72, 0.98) | 0.03 |
| Platelets at LD Chemotherapy |  | 1.00 | (1.00, 1.00) | 0.97 |
| CRP at LD Chemotherapy |  | 1.00 | (0.99, 1.02) | 0.98 |
| Ferritin at LD Chemotherapy |  | 1.25 | (1.05, 1.50) | 0.01 |
| LDH at LD chemotherapy |  | 1.32 | (0.90, 1.94) | 0.15 |
| CAR-HEMATOX Score |  | 1.24 | (1.03, 1.49) | 0.02 |
| CRS after CAR T-cell infusion |  | 1.72 | (0.73, 4.02) | 0.21 |
| Grade of CRS |  | 1.60 | (0.93, 2.76) | 0.088 |
| Received Treatment for CRS |  | 1.57 | (0.78, 3.18) | 0.21 |
| First CRS Therapy Given |  |  |  |  |
|  | Tocilizumab Only | Reference |  |  |
|  | Tocilizumab + Steroids | 0.77 | (0.39, 1.51) | 0.53 |
|  | Other | 1.10 | (0.20, 6.05) | 0.79 |
| Number Tocilizumab Doses Given |  | 1.07 | (0.76, 1.48) | 0.71 |
| ICANS after CAR T-cell infusion |  | 1.51 | (0.87, 2.63) | 0.14 |
| Grade of ICANS |  | 1.02 | (1.00, 1.05) | 0.11 |
| Treated for ICANS |  | 0.97 | (0.19, 5.05) | 0.97 |
| ICANS Treatment Given |  |  |  |  |
|  | Steroid Only | Reference |  |  |
|  | Tocilizumab + Steroids | 0.66 | (0.27, 1.65) | 0.43 |
|  | Other | 1.03 | (0.25, 4.30) | 0.75 |
| Febrile Neutropenia Following CART |  | 1.99 | (1.09, 3.63) | 0.02 |
| Neutropenic at Day 0 |  | 2.28 | (1.22, 4.27) | 0.010 |
| Lymphopenic at Day 0 |  | 0.70 | (0.22, 2.27) | 0.55 |
| Immunosuppressive Exposure |  | 1.03 | (0.59, 1.78) | 0.93 |
| Fluoroquinolone Duration |  | 1.05 | (0.99, 1.11) | 0.10 |
| Vancomycin Duration |  | 1.07 | (1.00, 1.15) | 0.057 |
| Carbapenem Duration |  | 1.07 | (1.01, 1.15) | 0.031 |
| Beta-lactam Duration |  | 1.03 | (1.00, 1.05) | 0.021 |

**Table S5.** Univariate logistic regression to evaluate association between exposures of interest and risk of infection within 90 days of chimeric antigen receptor T-cell infusion. CCI, Charlson Comorbidity Index; KPS, Karnofsky Performance Status; ECOG, Eastern Cooperative Oncology Group; CART, chimeric antigen receptor T-cell therapy; CR, Complete Response; PD, Progressive Disease; PR, Partial Response; SD, Stable Disease; ALL, Acute Lymphocytic Leukemia; DLBCL, Diffuse Large B-cell Lymphoma; NHL, Non-Hodgkin Lymphoma; HCT, Hematopoietic Cell Transplant; MUD, Matched Unrelated Donor; MRD, Matched Related Donor; CAR, Chimeric Antigen Receptor; LD, Lymphodepleting; ANC, Absolute Neutrophil Count; ALC, Absolute Lymphocyte Count; CRP, C-Reactive Protein; LDH, Lactate Dehydrogenase; CRS, cytokine release syndrome; ICANS, Immune Effector Cell-Associated Neurotoxicity Syndrome

| Variable |  | OR | UCL | p |
| --- | --- | --- | --- | --- |
| Age |  | 1.00 | (0.98, 1.01) | 1.00 |
| Sex |  |  |  |  |
|  | Male | Reference |  |  |
|  | Female | 0.88 | (0.49, 1.57) | 0.66 |
| Race |  |  |  |  |
|  | Caucasian | Reference |  |  |
|  | African descent | 3.18 | (1.28, 7.92) | 0.94 |
|  | Asian descent | 1.38 | (0.56, 3.45) | 0.96 |
|  | Pacific Islander | -- | -- | -- |
|  | Other | 2.18 | (0.98, 4.83) | 0.95 |
|  | Unknown | 0.62 | (0.08, 5.10) | 0.98 |
| Ethnicity |  |  |  |  |
|  | Non-Hispanic | Reference |  |  |
|  | Hispanic | 1.72 | (0.80, 3.69) | 0.27 |
|  | Unknown | 0.70 | (0.08, 5.97) | 0.57 |
| Study site |  |  |  |  |
|  | Duke | Reference |  |  |
|  | Stanford | 0.90 | (0.45, 1.79) | 0.66 |
|  | UNC | 1.05 | (0.43, 2.56) | 0.80 |
| CCI |  | 0.96 | (0.84, 1.10) | 0.58 |
| KPS |  | 0.99 | (0.96, 1.02) | 0.40 |
| ECOG |  | 1.00 | (0.98, 1.03) | 0.82 |
| Number of prior lines of chemotherapy |  | 1.09 | (0.95, 1.26) | 0.24 |
| Disease status Pre-CART |  |  |  |  |
|  | CR | Reference |  |  |
|  | PD | 0.77 | (0.08, 7.58) | 0.42 |
|  | PR | 0.66 | (0.06, 7.28) | 0.39 |
|  | SD | 0.69 | (0.06, 7.45) | 0.38 |
|  | Other | 3.00 | (0.08, 107.45) | 0.39 |
| Underlying Malignancy |  |  |  |  |
|  | ALL | Reference |  |  |
|  | DLBCL | 0.26 | (0.10, 0.71) | 0.99 |
|  | non-DLBCL NHL | 0.26 | (0.08, 0.85) | 0.99 |
| Prior HCT |  | 1.04 | (0.57, 1.89) | 0.90 |
| Type of Prior HCT |  |  |  |  |
|  | Autologous | Reference |  |  |
|  | Haploidentical | 3.22 | (0.49, 21.42) | 0.48 |
|  | MRD | 1.76 | (0.48, 6.46) | 0.87 |
|  | MUD | 2.42 | (0.40, 14.73) | 0.75 |
| CAR T-cell product |  |  |  |  |
|  | Anti-CD19 Trial | Reference |  |  |
|  | Tisagenlecleucel | 2.52 | (0.85, 7.46) | 0.95 |
|  | Brexucabtagene autoleucel | 1.01 | (0.27, 3.79) | 0.97 |
|  | Axicabtagene ciloleucel | 0.83 | (0.37, 1.87) | 0.97 |
|  | Other | -- | -- | -- |
| Lymphodepleting (LD) chemotherapy |  |  |  |  |
|  | Bendamustine | Reference |  |  |
|  | Fludarabine/ Bendamustine | -- | -- | -- |
|  | Fludarabine/ Cyclophosphamide | 0.50 | (0.05, 5.61) | 0.99 |
| Other |  | 1.20 | (0.07, 19.63) | 0.98 |
| ANC 30 Days Pre-LD |  | 0.91 | (0.83, 1.00) | 0.045 |
| ALC 30 Days Pre-LD |  | 0.91 | (0.83, 1.00) | 0.046 |
| ANC at LD Chemotherapy |  | 1.06 | (0.97, 1.16) | 0.17 |
| ALC at LD Chemotherapy |  | 1.07 | (0.87, 1.31) | 0.51 |
| Hemoglobin at LD Chemotherapy |  | 0.88 | (0.75, 1.02) | 0.10 |
| Platelets at LD Chemotherapy |  | 1.00 | (0.99, 1.00) | 0.19 |
| CRP at LD Chemotherapy |  | 1.05 | (0.94, 1.17) | 0.42 |
| Ferritin at LD Chemotherapy |  | 1.21 | (1.01, 1.46) | 0.04 |
| LDH at LD chemotherapy |  | 1.10 | (0.73, 1.64) | 0.66 |
| CAR-HEMATOX Score |  | 1.23 | (1.02, 1.48) | 0.03 |
| CRS after CAR T-cell infusion |  | 0.77 | (0.38, 1.58) | 0.47 |
| Grade of CRS |  | 1.49 | (0.85, 2.62) | 0.16 |
| Received Treatment for CRS |  | 2.37 | (1.06, 5.33) | 0.037 |
| First CRS Therapy Given |  |  |  |  |
|  | Tocilizumab Only | Reference |  |  |
|  | Tocilizumab + Steroids | 0.62 | (0.31, 1.25) | 0.31 |
|  | Other | 1.08 | (0.20, 5.95) | 0.71 |
| Number Tocilizumab Doses Given |  | 0.85 | (0.59, 1.22) | 0.37 |
| ICANS after CAR T-cell infusion |  | 1.32 | (0.76, 2.30) | 0.33 |
| Grade of ICANS |  | 1.01 | (0.98, 1.03) | 0.63 |
| Treated for ICANS |  | 2.17 | (0.26, 18.31) | 0.48 |
| ICANS Treatment Given |  |  |  |  |
|  | Steroids Only | Reference |  |  |
|  | Tocilizumab + Steroids | 1.46 | (0.61, 3.51) | 0.19 |
|  | Other | 0.38 | (0.05, 3.23) | 0.29 |
| Febrile neutropenia (FN) post-CAR T-cell infusion |  | 0.96 | (0.55, 1.69) | 0.89 |
| Neutropenic at Day 0 |  | 2.12 | (1.13, 3.99) | 0.020 |
| Lymphopenic at Day 0 |  | 0.68 | (0.21, 2.23) | 0.53 |
| Immunosuppressive Exposure |  | 1.16 | (0.66, 2.01) | 0.61 |
| Fluoroquinolone Duration |  | 1.01 | (0.99, 1.04) | 0.39 |
| Vancomycin Duration |  | 1.02 | (0.98, 1.05) | 0.39 |
| Carbapenem Duration |  | 1.02 | (0.98, 1.06) | 0.33 |
| Beta-lactam Duration |  | 1.00 | (0.99, 1.01) | 0.78 |

**Table S6.** Univariate logistic regression to evaluate association between exposures of interest and risk of bacterial infection within 1 year of chimeric antigen receptor T-cell infusion. CCI, Charlson Comorbidity Index; KPS, Karnofsky Performance Status; ECOG, Eastern Cooperative Oncology Group; CART, chimeric antigen receptor T-cell therapy; CR, Complete Response; PD, Progressive Disease; PR, Partial Response; SD, Stable Disease; ALL, Acute Lymphocytic Leukemia; DLBCL, Diffuse Large B-cell Lymphoma; NHL, Non-Hodgkin Lymphoma; HCT, Hematopoietic Cell Transplant; MUD, Matched Unrelated Donor; MRD, Matched Related Donor; CAR, Chimeric Antigen Receptor; LD, Lymphodepleting; ANC, Absolute Neutrophil Count; ALC, Absolute Lymphocyte Count; CRP, C-Reactive Protein; LDH, Lactate Dehydrogenase; CRS, cytokine release syndrome; ICANS, Immune Effector Cell-Associated Neurotoxicity Syndrome

| Variable |  | OR | UCL | p |
| --- | --- | --- | --- | --- |
| Age |  | 0.99 | (0.97, 1.00) | 0.99 |
| Sex |  |  |  |  |
|  | Male | Reference |  |  |
|  | Female | 1.52 | (0.89, 2.61) | 0.12 |
| Race |  |  |  |  |
|  | Caucasian | Reference |  |  |
|  | African descent | 0.58 | (0.16, 2.03) | 0.17 |
|  | Asian descent | 2.01 | (0.90, 4.49) | 0.26 |
|  | Pacific Islander | 1.92 | (0.17, 21.66) | 0.69 |
|  | Other | 1.69 | (0.77, 3.70) | 0.48 |
|  | Unknown | 1.10 | (0.22, 5.47) | 0.84 |
| Ethnicity |  |  |  |  |
|  | Non-Hispanic | Reference |  |  |
|  | Hispanic | 2.24 | (1.09, 4.61) | 0.28 |
|  | Unknown | 1.54 | (0.29, 8.12) | 0.98 |
| Study site |  |  |  |  |
|  | Duke | Reference |  |  |
|  | Stanford | 1.18 | (0.60, 2.32) | 0.48 |
|  | UNC | 0.93 | (0.37, 2.31) | 0.69 |
| CCI |  | 0.97 | (0.86, 1.10) | 0.64 |
| KPS |  | 1.00 | (0.97, 1.02) | 0.78 |
| ECOG |  | 1.01 | (0.99, 1.04) | 0.17 |
| Number of prior lines of chemotherapy |  | 1.05 | (0.91, 1.21) | 0.47 |
| Disease status Pre-CART |  |  |  |  |
|  | Non-Responder | Reference |  |  |
|  | Responder | 0.61 | (0.26, 1.44) | 0.26 |
| Underlying Malignancy |  |  |  |  |
|  | ALL | Reference |  |  |
|  | DLBCL | 0.65 | (0.22, 1.92) | 0.97 |
|  | non-DLBCL NHL | 0.86 | (0.26, 2.89) | 0.98 |
| Prior HCT |  | 1.08 | (0.61, 1.91) | 0.78 |
| Type of Prior HCT |  |  |  |  |
|  | Autologous | Reference |  |  |
|  | Haploidentical | 2.67 | (0.41, 17.52) | 0.77 |
|  | MRD | 1.00 | (0.25, 4.03) | 0.19 |
|  | MUD | 8.00 | (1.33, 48.15) | 0.06 |
| CAR T-cell product |  |  |  |  |
|  | Anti-CD19 Trial | Reference |  |  |
|  | Tisagenlecleucel | 0.65 | (0.20, 2.11) | 0.28 |
|  | Brexucabtagene autoleucel | 1.51 | (0.48, 4.74) | 0.54 |
|  | Axicabtagene ciloleucel | 0.68 | (0.32, 1.42) | 0.15 |
|  | Other | 2.58 | (0.15, 44.70) | 0.46 |
| Lymphodepleting (LD) chemotherapy |  |  |  |  |
|  | Bendamustine-containing Regimen | Reference |  |  |
|  | Fludarabine/ Cyclophosphamide | 2.10 | (0.25, 17.34) | 0.64 |
|  | Other | 2.33 | (0.17, 32.58) | 0.63 |
| ANC 30 Days Pre-LD |  | 0.99 | (0.92, 1.06) | 0.74 |
| ALC 30 Days Pre-LD |  | 1.01 | (0.94, 1.09) | 0.74 |
| ANC at LD Chemotherapy |  | 1.00 | (0.91, 1.09) | 0.95 |
| ALC at LD Chemotherapy |  | 0.93 | (0.77, 1.12) | 0.43 |
| Hemoglobin at LD Chemotherapy |  | 1.01 | (0.87, 1.16) | 0.94 |
| Platelets at LD Chemotherapy |  | 1.00 | (1.00, 1.00) | 0.42 |
| CRP at LD Chemotherapy |  | 1.04 | (0.93, 1.15) | 0.51 |
| Ferritin at LD Chemotherapy |  | 1.05 | (0.90, 1.23) | 0.51 |
| LDH at LD chemotherapy |  | 1.15 | (0.79, 1.67) | 0.48 |
| CAR-HEMATOX Score |  | 1.15 | (0.96, 1.38) | 0.12 |
| CRS after CAR T-cell infusion |  | 1.03 | (0.49, 2.13) | 0.95 |
| Grade of CRS |  | 0.93 | (0.55, 1.59) | 0.80 |
| Received Treatment for CRS |  | 1.16 | (0.60, 2.25) | 0.658 |
| First CRS Therapy Given |  |  |  |  |
|  | Tocilizumab Only | Reference |  |  |
|  | Tocilizumab + Steroids | 0.52 | (0.26, 1.04) | 0.96 |
|  | Other | -- | -- | -- |
| Number Tocilizumab Doses Given |  | 1.36 | (0.99, 1.88) | 0.06 |
| ICANS after CAR T-cell infusion |  | 1.45 | (0.85, 2.46) | 0.17 |
| Grade of ICANS |  | 1.01 | (0.98, 1.03) | 0.64 |
| Treated for ICANS |  | 2.46 | (0.29, 20.73) | 0.41 |
| ICANS Treatment Given |  |  |  |  |
|  | Steroids only | Reference |  |  |
|  | Tocilizumab + Steroids | 1.21 | (0.52, 2.85) | 0.86 |
|  | Other | 1.23 | (0.29, 5.16) | 0.88 |
| Febrile neutropenia (FN) post-CAR T-cell infusion |  | 1.50 | (0.86, 2.63) | 0.16 |
| Neutropenic at Day 0 |  | 1.90 | (1.03, 3.53) | 0.041 |
| Lymphopenic at Day 0 |  | 1.19 | (0.33, 4.35) | 0.79 |
| Immunosuppressive Exposure |  | 0.92 | (0.54, 1.56) | 0.75 |
| Fluoroquinolone Duration |  | 1.00 | (0.96, 1.05) | 0.87 |
| Vancomycin Duration |  | 1.02 | (0.99, 1.05) | 0.15 |
| Carbapenem Duration |  | 1.05 | (1.00, 1.09) | 0.040 |
| Beta-lactam Duration |  | 1.01 | (1.00, 1.02) | 0.06 |

**Table S7.** Univariate logistic regression to evaluate association between exposures of interest and risk of viral infection within 1 year of chimeric antigen receptor T-cell infusion. CCI, Charlson Comorbidity Index; KPS, Karnofsky Performance Status; ECOG, Eastern Cooperative Oncology Group; CART, chimeric antigen receptor T-cell therapy; CR, Complete Response; PD, Progressive Disease; PR, Partial Response; SD, Stable Disease; ALL, Acute Lymphocytic Leukemia; DLBCL, Diffuse Large B-cell Lymphoma; NHL, Non-Hodgkin Lymphoma; HCT, Hematopoietic Cell Transplant; MUD, Matched Unrelated Donor; MRD, Matched Related Donor; CAR, Chimeric Antigen Receptor; LD, Lymphodepleting; ANC, Absolute Neutrophil Count; ALC, Absolute Lymphocyte Count; CRP, C-Reactive Protein; LDH, Lactate Dehydrogenase; CRS, cytokine release syndrome; ICANS, Immune Effector Cell-Associated Neurotoxicity Syndrome

| Variable |  | OR | UCL | p |
| --- | --- | --- | --- | --- |
| **Infection within 90 days of CAR T-cell Infusion** | | | | |
| Age |  | 1.00 | (0.98, 1.02) | 0.89 |
| Underlying Malignancy |  |  |  |  |
|  | ALL | Reference |  |  |
|  | DLBCL | 1.05 | (0.24, 4.56) | 1.00 |
|  | non-DLBCL NHL | 1.10 | (0.23, 5.26) | 0.89 |
| Neutropenic at Day 0 |  | 1.80 | (0.91, 3.57) | 0.09 |
| Carbapenem duration |  | 1.05 | (0.97, 1.13) | 0.24 |
| Beta-lactam duration |  | 1.02 | (1.00, 1.04) | 0.10 |
| **Infection after 90 days following CAR T-cell Infusion** | | | | |
| Age |  | 0.99 | (0.97, 1.02) | 0.55 |
| Underlying Malignancy |  |  |  |  |
|  | ALL | Reference |  |  |
|  | DLBCL | 0.53 | (0.15, 1.93) | 0.71 |
|  | non-DLBCL NHL | 0.38 | (0.09, 1.53) | 0.16 |
| Neutropenic at Day 0 |  | 0.92 | (0.46, 1.81) | 0.80 |
| Lymphopenic at Day 0 |  | 0.98 | (0.28, 3.38) | 0.97 |
| CRS Grade |  |  |  |  |
|  | Grade <2 | Reference |  |  |
|  | Grade ≥2 | 0.75 | (0.43, 1.32) | 0.32 |
| ICANS Grade |  |  |  |  |
|  | Grade 0 | Reference |  |  |
|  | Grade 1-2 | 1.54 | (0.79, 2.98) | 0.85 |
|  | Grade 3-4 | 2.10 | (1.03, 4.27) | 0.12 |
| **Bacterial Infection within 1 year of CAR T-cell Infusion** | | | | |
| Age |  | 1.02 | (0.99, 1.04) | 0.22 |
| Underlying Malignancy |  |  |  |  |
|  | ALL | Reference |  |  |
|  | DLBCL | 0.25 | (0.06, 0.98) | 0.12 |
|  | non-DLBCL NHL | 0.23 | (0.05, 1.02) | 0.12 |
| ANC 30 Days Pre-LD |  | 1.00 | (0.83, 1.21) | 1.00 |
| ALC 30 Days Pre-LD |  | 0.91 | (0.75, 1.11) | 0.36 |
| Neutropenic at Day 0 |  | 1.85 | (0.93, 3.67) | 0.08 |
| **Viral Infection within 1 year of CAR T-cell Infusion** | | | | |
| Age |  | 0.99 | (0.97, 1.01) | 0.24 |
| Underlying Malignancy |  |  |  |  |
|  | ALL | Reference |  |  |
|  | DLBCL | 1.30 | (0.34, 4.96) | 1.00 |
|  | non-DLBCL NHL | 1.70 | (0.41, 7.04) | 0.38 |
| Neutropenic at Day 0 |  | 1.94 | (1.02, 3.68) | 0.04 |

**Table S8.** Multivariate logistic regression to evaluate associations between exposures of interest and risk of infection within 90 days of CART, after 90 days post-CART, bacterial infection, and viral infection within 1 year of chimeric antigen receptor T-cell infusion. ALL, Acute Lymphocytic Leukemia; DLBCL, Diffuse Large B-cell Lymphoma; NHL, Non-Hodgkin Lymphoma; CRS, cytokine release syndrome; ICANS, Immune Effector Cell-Associated Neurotoxicity Syndrome; ANC, Absolute Neutrophil Count; ALC, Absolute Lymphocyte Count

DEFINITION OF INFECTION

Infection events during the first year following CAR T-cell infusion were defined as those meeting any of the following criteria:

1. Viral Infection
   1. CMV
      1. Detection of CMV DNA in the blood leading to treatment
      2. CMV syndrome
         1. Probable: Requires detection of CMV DNA via plasma PCR testing AND at least 2 of the following:
            1. Fever ≥38°C at least 2 days.
            2. New or increased malaise (toxicity grade 2) or new or increased fatigue (toxicity grade 3) (National Cancer Institute: Common Terminology Criteria for Adverse Events, version 5.0).
            3. Leukopenia or neutropenia on 2 separate measurements at least 24 hours apart, defined as a WBC of <3500 cells/µL, if the WBC count prior to the development of clinical symptoms was ≥4000 cells/µL, or a WBC decrease of >20%, if the WBC count prior to the development of clinical symptoms was <4000 cells/µL. The corresponding neutrophil counts are <1500 cells/µL or a decrease of >20% if the neutrophil count before the onset of symptoms was <1500 cells/µL.
            4. Greater than or equal to 5% atypical lymphocytes.
            5. Thrombocytopenia defined as a platelet count of <100000 cells/µL if the platelet count prior to the development of clinical symptoms was ≥115000 cells/µL or a decrease of >20% if the platelet count prior to the development of clinical symptoms was <115000 cells/µL.
            6. Elevation of hepatic aminotransferases (alanine aminotransferase or aspartate aminotransferase) to 2 times the upper limit of normal.
         2. Proven: Not Applicable
      3. CMV disease (see definitions below)
         1. Pneumonia
            1. Probable: clinical symptoms and/or signs of pneumonia AND detection of CMV by viral isolation, rapid culture of BAL fluid, or the quantitation of CMV DNA in BAL fluid
            2. Proven: clinical symptoms and/or signs of pneumonia (e.g., new infiltrates on imaging, hypoxia, tachypnea, and/or dyspnea) AND CMV documented in lung tissue by virus isolation, rapid culture, histopathology, IHC, or DNA hybridization techniques.
         2. Gastrointestinal
            1. Probable: upper and/or lower GI symptoms AND CMV documented in tissue by histopathology, virus isolation, rapid culture, IHC, or DNA hybridization techniques (without the requirement for macroscopic mucosal lesions).
            2. Proven: upper and/or lower gastrointestinal (GI) symptoms AND macroscopic mucosal lesions AND CMV documented in tissue.
         3. Hepatitis
            1. Probable: not a recommended category.
            2. Proven: abnormal liver function tests plus CMV documented in tissue by histopathology, IHC, virus isolation, rapid culture, or DNA hybridization techniques AND absence of other documented cause of hepatitis.
         4. Retinitis
            1. Probable: not a recommended category.
            2. Proven: typical ophthalmological signs judged by an ophthalmologist experienced with the diagnosis of CMV retinitis. If the presentation is atypical or an experienced ophthalmologist is not available, it is recommended that the diagnosis be supported by CMV documented in vitreous fluid by NAT (such as PCR).
         5. Encephalitis/Ventriculitis:
            1. Probable: CNS symptoms AND detection of CMV in CSF without visible contamination of blood (“bloody tap”) AND abnormal imaging results or evidence of encephalitis on electroencephalography.
            2. Proven: requires CNS symptoms AND detection of CMV in CNS tissue by virus isolation, rapid culture, IHC, in situ hybridization, or (preferably) quantitative PCR.
         6. Nephritis
            1. Probable: not a recommended category.
            2. Proven: detection of CMV by virus isolation, rapid culture, IHC, or in situ hybridization in a kidney allograft biopsy specimen obtained from a patient with renal dysfunction AND identification of histologic features of CMV infection.
         7. Cystitis
            1. Probable: not a recommended category.
            2. Proven: detection of CMV by virus isolation, rapid culture, IHC, or in situ hybridization in a bladder biopsy specimen obtained from a patient with cystitis AND identification of conventional histologic features of CMV infection.
         8. Myocarditis
            1. Probable: not a recommended category.
            2. Proven: detection of CMV by virus isolation, rapid culture, IHC, or in situ hybridization in a heart biopsy specimen obtained from a patient with myocarditis AND identification of conventional histologic features of CMV infection.
         9. Pancreatitis
            1. Probable: not a recommended category.
            2. Proven: detection of CMV by virus isolation, rapid culture, IHC, or in situ hybridization in a pancreatic biopsy specimen obtained from a patient with pancreatitis AND identification of conventional histologic features of CMV infection.
         10. Other End-Organ Disease
             1. Presence of compatible symptoms and signs AND documentation of CMV by biopsy by virus isolation, rapid culture, IHC, or DNA hybridization in biopsy material.
   2. Herpes Simplex Virus (HSV)
      1. Orolabial or Genital Infection
         1. Presence of vesicular-type lesions in orolabial or genital areas. While virological confirmation is preferred, it is not essential to confirm a clinical diagnosis.
      2. Tissue-invasive disease
         1. Presence of organ dysfunction in the absence of other documented cause AND detection of HSV in biopsy tissue by culture, DNA/RNA-based assay, IHC or *in situ* hybridization
   3. Varicella zoster virus (VZV)
      1. Cutaneous Infection
         1. Probable*:* presence of crops of vesicular lesions on an erythematous base. This may be primary infection (in a previously naïve host) or reactivation. Dermatomal distribution is sufficient to diagnose probable reactivation disease of VZV (zoster, shingles).
         2. Proven: presence of typical lesions with isolation of VZV, demonstration of VZV antigen by direct fluorescent antibody or by DNA/RNA-based tests from a lesion.
      2. Tissue-invasive Disease
         1. Probable: Not Applicable
         2. Proven: presence of organ dysfunction in the absence of another documented cause in association with detection of VZV in biopsy tissue by culture, DNA/RNA-based assay, IHC analysis or *in situ* hybridization.
   4. Human Herpes Virus 6 (HHV6)
      1. Detection of HHV6 DNA in the blood leading to treatment
      2. Detection of HHV6 DNA in the blood or cerebral spinal fluid (CSF) with
         1. A clinical syndrome consistent with HHV6 encephalitis (i.e. altered mental status, psychosis, seizures, acute cerebellar ataxia, or focal neurological deficits) or magnetic resonance imaging (MRI) findings consistent with HHV6 encephalitis.
   5. BK polyomavirus
      1. Clinically significant BK DNAemia
         1. Plasma load > 4 log10 copies/mL (with subsequent increase in serial measurements)
      2. Proven PyVAN
         1. Renal biopsy demonstrating cytopathic changes of tubular epithelial cells in the allograft tissue AND confirmed by IHC or *in situ* hybridization
   6. JC Polyomavirus
      1. Proven PML
         1. Appropriate imaging and clinical features AND PCR detection of JCV DNA in the cerebrospinal fluid OR brain biopsy with demyelination, bizarre astrocytes, and enlarged oligodendroglial nuclei AND detection of JCV protein by IHC, electron microscopy or tissue PCR.
      2. Probable PML
         1. JCV DNA detected in the CSF AND either imaging or clinical features present OR brain biopsy with demyelination, bizarre astrocytes, and enlarged oligodendroglial nuclei only
   7. Parvovirus-B19 (PVB19)
      1. PVB19 Infection
         1. Molecular detection (PCR) of PBV19 in the blood in the presence of anemia with clinical symptoms or bone marrow biopsy findings consistent with the diagnosis.
      2. PVB19 Organ-Invasive Disease
         1. Concomitant with PVB19 molecular detection in blood, organ-specific findings (as indicated by biochemical markers, pathological findings, or radiographic imaging). Must meet the following criteria:
            1. Detection of PVB19 by PCR or other methodology in tissue specimens
            2. Response to PVB19-directed therapy
            3. Absence of other infectious or noninfectious processes that could explain the organ-specific findings
   8. Epstein-Barr Virus (EBV)
      1. Clinically Significant EBV Viremia
         1. Detection of EBV in whole blood in > 2 consecutive samples at a value > 10000 IU/mL
      2. EBV Disease
         1. Presence of active EBV viremia with symptoms or signs attributable to the virus.
   9. Adenovirus
      1. Attributable signs and symptoms without an alternative diagnosis AND detection of adenovirus in biopsy specimens via IHC, culture, antigen detection or PCR. PCR testing can also be performed in body fluids, including blood, BAL, and CSF. If at least two organs are involved (excluding viremia), the disease should be considered disseminated.
   10. SARS-CoV-2
       1. Laboratory-confirmed SARS-CoV-2 by RT-PCR of an upper or lower respiratory sample.
   11. Other Community- Acquired Respiratory Viruses
       1. Upper Respiratory Tract Infection
          1. A positive result for a community respiratory virus from a respiratory specimen AND the presence of clinical signs/symptoms of a respiratory infection, including fever, rhinorrhea, nasal congestion, pharyngitis, and/or headache AND the absence of lower respiratory tract symptoms and/or new infiltrates on chest imaging.
       2. Lower Respiratory Tract Infection
          1. A positive result for a community respiratory virus from a respiratory specimen AND The presence of clinical signs/symptoms of a lower respiratory tract infection, including cough, shortness of breath, and/or hypoxia AND The presence of a new infiltrate on chest imaging.
2. Bacterial Infection
   1. Bloodstream Infection
      1. Non-commensal Organism
         1. A recognized bacterial or fungal pathogen, not included on the National Healthcare Safety Network (NHSN) common commensal list, identified from one or more blood cultures
         2. At least one of the following signs or symptoms: fever (>38.0^o^C), chills, or hypotension AND the same NHSN common commensal is identified from two or more blood cultures collected on separate occasions.
   2. Lung Infection
      1. Pneumonia
         1. Isolation of a bacterial organism from an upper or lower respiratory specimen AND imaging test evidence AND signs/symptoms as defined below:
            1. Imaging test evidence: chest imaging with at least one of the following NEW or PROGRESSIVE findings: infiltrate, consolidation, and/or cavitation.
            2. Signs/symptoms: at least one of the following:

Fever (>38.0°C or >100.4°F)

Leukopenia (≤4000 WBC/mm^3^) or leukocytosis (≥12,000 WBC/mm^3^)

New onset of purulent sputum or change in character of sputum or increased respiratory secretions, or increased suctioning requirements

New onset or worsening cough, or dyspnea, or tachypnea

Rales or bronchial breath sounds

Worsening gas exchange (e.g., O2 desaturation, increased oxygen requirements, or increased ventilator demand)

- - 1. Eye, ear, nose, throat infections
       1. Eye infection (other than conjunctivitis)
          1. Patient has organism(s) identified from the anterior or posterior chamber or vitreous fluid by a culture or non-culture based microbiologic testing method.
       2. Pharyngitis, laryngitis, epiglottitis
          1. Organism(s) identified from the upper respiratory site AND at least two of the following signs or symptoms: fever (>38.0°C), erythema of pharynx, sore throat, cough, hoarseness, or purulent exudate in the throat
       3. Ear/Mastoid Infections
          1. Otitis externa

Patient has organism(s) identified from purulent drainage from the ear canal by a culture or relevant non-culture based microbiologic testing method.

- - - - 1. Otitis media

Patient has organism(s) identified from fluid from the middle ear obtained during an invasive procedure (for example, tympanocentesis) by a culture or relevant non-culture based microbiologic testing method.

- - - - 1. Otitis interna

Patient has organism(s) identified from fluid from the inner ear obtained during an invasive procedure by a culture or relevant non-culture based microbiologic testing method.

- - - - 1. Mastoiditis

Patient has organism(s) identified from fluid or tissue from the mastoid by a culture or relevant non-culture based microbiologic testing method.

- - - 1. Sinusitis
         1. Patient has organism(s) identified from fluid or tissue from the sinus cavity obtained during an invasive procedure by a culture or relevant non-culture based microbiologic testing method.
  1. Gastrointestinal Infections
     1. Gastroenteritis (excluding *Clostridioides difficile* infection)
        1. Patient has at least two of the following signs or symptoms: acute onset of diarrhea (liquid stools for > 12 hours) and no likely noninfectious cause, nausea, vomiting, abdominal pain, fever (>38.0°C), or headache AND at least one of the following:
           1. Enteric pathogen identified from stool or rectal swab by a culture or relevant non-culture based microbiologic testing method
           2. Enteric pathogen is detected by microscopy on stool
     2. *Clostridioides difficile* infection
        1. Positive laboratory diagnostic test result [i.e. NAAT for *C. difficile* toxin B gene or a positive stool test for *C. difficile* toxin(s)] AND unformed stools or evidence of megacolon, evidence of pseudomembranes demonstrated by endoscopy or histopathology, or severe ileus OR application of *C. difficile* directed therapy.
     3. Intraabdominal Infection (excluding *Clostridioides difficile* infection and gastroenteritis)
        1. Patient must have one of the following:
           1. Organism(s) identified from an abscess or from purulent material from intraabdominal space by a culture
           2. Patient has at least one of the following: fever (>38.0°C), hypotension, nausea, vomiting, abdominal pain or tenderness, elevated transaminase level(s), or jaundice AND organism(s) identified from blood culture(s) AND imaging test evidence suggestive of infection (e.g., ultrasound, CT scan, MRI, ERCP, radiolabel scans [gallium, technetium, etc.] or on abdominal x-ray), which if equivocal is supported by clinical correlation, specifically, physician documentation of antimicrobial treatment for intraabdominal infection.
  2. Genitourinary
     1. Catheter-associated Urinary Tract Infection
        1. Must meet all of the following criteria:
           1. Patient had an indwelling urinary catheter that had been in place for more than 2 consecutive days on the date of event AND was either present for any portion of the calendar day on the date of event OR removed the day before the date of event
           2. Patient has at least one of the following signs or symptoms:

fever (>38.0°C)

suprapubic tenderness

costovertebral angle pain or tenderness

urinary urgency

urinary frequency

dysuria

- - - - 1. Patient has a urine culture with no more than two species of organisms identified, at least one of which is a bacterium of ≥10^5^ CFU/ml
    1. Non-catheter-associated Urinary Tract Infection (including pyelonephritis)
       1. Must meet all of the following criteria:
          1. Patient has at least one of the following signs or symptoms:

fever (>38.0°C)

suprapubic tenderness

costovertebral angle pain or tenderness

urinary urgency

urinary frequency

dysuria

- - - - 1. Patient has a urine culture with no more than two species of organisms identified, at least one of which is a bacterium of ≥10^5^ CFU/ml.
    1. Genitourinary Infection (infection of the kidney, ureter, bladder, urethra, or perinephric space, excluding urinary tract infection and pyelonephritis
       1. Must meet at least one of the following criteria
          1. Patient has organism(s) identified from fluid (not urine) or tissue from the affected site by a culture.
          2. Patient has an abscess or other evidence of infection on gross anatomical exam, during invasive procedure, or on histopathologic exam.
          3. Patient has one of the following signs or symptoms: fever (>38.0°C), localized pain or tenderness AND at least one of the following:

Purulent drainage from the affected site.

Organism(s) identified from blood AND imaging test evidence suggestive of infection, which, if equivocal, is supported by clinical correlation, specifically, physician documentation of antimicrobial treatment for urinary system infection.

- 1. Skin and Soft Tissue Infections
     1. Skin Infection (skin/subcutaneous)
        1. Skin infections must meet at least one of the following criteria in addition to a pathogen identified by microbiologic testing from the affected site:
           1. Patient has at least one of the following: purulent drainage, pustules, and/or boils (excluding acne)
           2. Patient has at least two of the following localized signs or symptoms: pain or tenderness, swelling, erythema, and/or heat.
     2. Soft Tissue Infection (muscle and/or fascia)
        1. Soft tissue infections must meet at least one of the following criteria in addition to a pathogen identified by microbiologic testing from the affected site:
           1. Patient has purulent drainage at the affected site
           2. Patient has an abscess or other evidence of infection on gross anatomic or histopathologic exam
  2. Cardiovascular Infections
     1. Myocarditis or pericarditis
        1. Patient has organism(s) identified from pericardial tissue or fluid by a culture or non-culture based microbiologic testing method
     2. Infective Endocarditis
        1. Satisfaction of modified Duke criteria for Definite or Possible infective endocarditis.^29^
